# The Prominent Deck B Phenomenon in Schizophrenia: An Empirical Study on Iowa Gambling Task

**DOI:** 10.3389/fpsyg.2021.619855

**Published:** 2021-09-03

**Authors:** Mei Xu, We-Kang Lee, Chih-Hung Ko, Yao-Chu Chiu, Ching-Hung Lin

**Affiliations:** ^1^Department of Psychology, Kaohsiung Medical University, Kaohsiung, Taiwan; ^2^School of Psychiatry, Faculty of Medicine, University of New South Wales, Sydney, NSW, Australia; ^3^Sleep Center, Shin Kong Wu Ho-Su Memorial Hospital, Taipei, Taiwan; ^4^Department of Psychology, Soochow University, Taipei, Taiwan; ^5^Department of Psychiatry, Kaohsiung Medical University Hospital, Kaohsiung, Taiwan; ^6^Department of Psychiatry, Kaohsiung Municipal Siaogang Hospital, Kaohsiung, Taiwan; ^7^College of Medicine, Graduate Institute of Medicine, Kaohsiung Medical University, Kaohsiung, Taiwan; ^8^Research Center for Non-linear Analysis and Optimization, Kaohsiung Medical University, Kaohsiung, Taiwan

**Keywords:** Iowa Gambling Task, prominent deck B phenomenon, gain-loss frequency, schizophrenia, expected value, decision making

## Abstract

**Background:** The Iowa Gambling Task (IGT) was established to evaluate emotion-based decision-making ability under uncertain circumstances in clinical populations, including schizophrenia (Sz). However, there remains a lack of stable behavioral measures regarding discrimination for decision-making performance in IGT between schizophrenic cases and healthy participants. None of the Sz-IGT studies has specifically verified the prominent deck B (PDB) phenomenon gradually revealed in other populations. Here, we provided a global review and empirical study to verify these Sz-IGT issues.

**Methods:** Seeking reliable and valid behavioral measures, we reviewed 38 studies using IGT to investigate decision-making behavior in Sz groups. The IGT, the Wisconsin Card Sorting Test (WCST), and clinical symptoms evaluations were administered to 61 schizophrenia or schizoaffective cases diagnosed by psychiatrists and 62 demographically matched healthy participants.

**Results:** There were no valid behavioral measures in IGT that could significantly identify the decision-making dysfunction of Sz. However, Sz cases, on average, made more choices from disadvantageous deck B relative to other decks, particularly in the later learning process (block 3–5). Compared to the control group, the Sz group was more impaired on the WCST. The high-gain frequency decks B and D showed significant correlations with WCST but no correlation between clinical symptoms and IGT/WCST.

**Conclusions:** Gain–loss frequency (GLF) has a dominant and stable impact on the decision-making process in both Sz and control groups. PDB phenomenon is essentially challenging to be observed on the ground of the expected value (EV) viewpoint approach on the IGT in both populations. Consequently, caution should be exercised when launching the IGT to assess the decision-making ability of Sz under a clinical scenario.

## Introduction

Schizophrenia (Sz) remains a chronic, severe, and complicated psychiatric disorder with positive symptoms (i.e., hallucinations, delusions, disorganized thinking, and disorganized behaviors) and negative symptoms (i.e., blunted affect, alogia, asociality, anhedonia, and avolition) (American Psychiatric Association, 2013). It is generally accepted that Sz cases have impaired learning and rewarding systems (Waltz and Gold, 2007; Saperia et al., 2019; Woodrow et al., 2019), dysfunctional emotion processing (Trémeau, 2006), and decision-making deficits in goal-directed behavior (Gold et al., 2008; Saperia et al., 2019). Patients with Sz are impaired in flexible and value-based decision-making, particularly in changing and volatile environments (Waltz and Gold, 2007). Consistent evidence supports that Sz displays disrupted reward anticipation and reinforcement learning on behavioral and neural levels (Dayan and Daw, 2008). In Sz, decision-making dysfunction has been related to both positive and negative symptoms (Sterzer et al., 2019). The “jumping-to-conclusion” (JTC) bias refers to a tendency to make hasty decisions without sufficient information, which is related to positive symptoms, particularly delusions in Sz (Evans et al., 2015). Alternations in reward processing associated with negative symptoms may lead to inappropriate evaluation and analysis of long-term rewards guiding short-term decision-making behavior (Gold et al., 2008; Maia and Frank, 2017).

Review and meta-analysis studies have provided behavioral evidence that Sz has impaired reward-based decision-making process (Brown et al., 2015; Betz et al., 2018). In terms of the evidence from neuroimaging studies, Sz cases show hypofrontality with fewer activations in the frontal cortex, including dorsolateral frontal cortex (DLPFC) assessed with the Wisconsin Card Sorting Test (WCST) (Riehemann et al., 2001). Sz cases typically present a higher number of perseverative errors in WCST that is negatively correlated with DLPFC in Magnetic Resonance Imaging (MRI) and is related to reduced DLPFC activation in Functional Magnetic Resonance Imaging (fMRI), suggesting a deficit in switching and inhibitory functions (Seidman et al., 1994; Riehemann et al., 2001). The orbitofrontal cortex (OFC)/ ventromedial prefrontal cortex (VMPFC) associated with decision-making performance, reversal learning, and devaluation ability is also suggested to be impaired in Sz cases (Kringelbach, 2005; Nakamura et al., 2007). Previous studies using the Iowa gambling task (IGT), involving the interaction of “hot” affective signals and “cold” rational processing, highlighted that emotion plays a critical role in the decision-making process related to VMPFC (Bechara et al., 1997, 2005; Chiu et al., 2018). This laboratory task was developed to mimic daily life context and assess individual emotion-based decision-making behavior under ambiguity. It has been used with diverse clinical populations [i.e., Sz, substance addiction, pathological gambling, anorexia nervosa, obesity, chronic pain, aggression disorders, affective disorders, Huntington's disease, obsessive–compulsive disorder (OCD), attention**-**deficit/hyperactivity disorder (ADHD)] (Bechara, 2007, 2016).

### Iowa Gambling Task

Initially, Bechara et al. developed the IGT in 1994, aiming to verify the somatic marker hypothesis (SMH). SMH assumed that people had intact somatic marker systems that could assist them in making decisions beneficial to long-term outcomes under uncertain situations. Given the support of neural circuits of healthy somatic markers, participants could achieve a final win (total amount of money > 0) in IGT. Conversely, if this system was impaired (e.g., in VMPFC-impaired patients), individuals could not generate behavior that could avoid losses and tended to ignore long-term profits. This suggested that participants were easily affected by immediate losses and gains and neglected long-term benefits, which ultimately ended with a final loss in the IGT (Bechara et al., 1994, 1997, 1999).

The original IGT consisted of four decks (see Supplementary Figure 1). Participants had 100 trials for selection and they were free to choose from four decks. At the initiation of the experiment, participants had a loan of $2,000 from the bank and were informed instantly about the amount of money gained or lost after each choice. Participants were informed to try their best to determine the winning strategy and to maximize the money they gained. Of the four decks, decks A and B were regarded as disadvantageous decks because of the negative expected value (EV) ($-250) based on every 10 cards. By contrast, decks C and D were advantageous decks because of the positive EV (*$*+250). In terms of gain–loss frequency (GLF) of IGT, decks A and C had similar gain–loss structure: 10 gains and 5 losses per 10 cards; decks B and D shared a similar high winning frequency of 10 gains and 1 loss per 10 cards (see Supplementary Table 2).

### Expected Value Viewpoint: IGT and Net Score

Bechara et al. (1994) believed that typical VMPFC-impaired patients had intact intellect and problem-solving abilities, but they could not learn the concept of EV lacking intact somatic marker systems and chose more cards from decks A and B with negative EV in IGT. In contrast, with the guidance of somatic marker systems, healthy subjects who could gradually learn the concept of EV and were sensitive to future outcomes could make more selections from deck C and deck D with positive EV in IGT (Bechara and Damasio, 2002). In 1994, Bechara et al. compared 44 controls and six VMPFC-impaired patients and showed that the healthy participants picked more cards from advantageous decks, whereas the VMPFC-impaired patients selected more cards from the disadvantageous decks.

Bechara et al. developed the net score, which took the difference between choices from advantageous decks and disadvantageous decks [(C+D) - (A+B)] as a behavioral measure of whether participants were sensitive to future outcomes (Bechara et al., 1994). Most subsequent studies used this behavioral measure to probe decision-making patterns in Sz and control groups from the viewpoint of EV (Shurman et al., 2005; Kester et al., 2006; Sevy et al., 2007; Premkumar et al., 2008; Lee et al., 2009; Shirayama et al., 2010; Nestor et al., 2014; Matsuzawa et al., 2015; Stratta et al., 2015; Kim et al., 2016; Pedersen et al., 2017; Glick et al., 2021). However, interestingly, the evidence from Sz-IGT literature was somewhat inconclusive in relation to EV. Some studies revealed that Sz cases preferred disadvantageous decks relative to advantageous decks (Beninger et al., 2003; Ritter et al., 2004; Shurman et al., 2005; Kester et al., 2006; Lee et al., 2007; Nakamura et al., 2007; Premkumar et al., 2008; Yip et al., 2009; Wasserman et al., 2012; Brown et al., 2013; Nestor et al., 2014; Matsuzawa et al., 2015; Kim et al., 2016). Other studies suggested that Sz cases and healthy subjects performed equally on the net score in IGT (Wilder et al., 1998; Bark et al., 2005; Evans et al., 2005; Rodríguez-Sánchez et al., 2005; Turnbull et al., 2006; Martino et al., 2007; Sevy et al., 2007; González-Blanch et al., 2008; Shirayama et al., 2010; Choi et al., 2011; Carvalho et al., 2012; Ayesa-Arriola et al., 2013; Premkumar et al., 2015; Pedersen et al., 2017; Glick et al., 2021) (See Supplementary Table 1).

The contrasting results in Sz-IGT studies were likely due to the heterogeneity of characteristics of participants and methodological approaches (e.g., disparate outcome measures of IGT). Types of antipsychotic treatments (Beninger et al., 2003), doses of medication (Betz et al., 2018), diagnoses (Betz et al., 2018), clinical symptoms (Betz et al., 2018), intelligence level (Betz et al., 2018), age (Carvalho et al., 2012), gender (Singh et al., 2020), and education (Evans et al., 2004) affect decision-making performance in Sz cases. However, no conclusions based on the above factors could be made to reach a consensus. Among all the potential factors, the scoring approach is a key factor. Across 38 Sz-IGT studies, there were 15 types of outcome measures (Supplementary Figure 1): most studies (84.2%) used net score [(C+D) - (A+B)]; the four-deck format (47.4%) was also preferred by Sz-IGT studies; an equal number of studies employed advantageous decks (C+D) (15.8%) and disadvantageous decks (A+B) (15.8%); three studies used GLF measures [(B+D) - (A+C), B+D, or A+C]; and other scoring approaches accounted for fewer than 10 studies. Various outcome measures were developed, even where the net score was taken as the primary outcome measure.

IGT reviews have suggested that the four-deck format (scoring the number of selections from deck A, B, C, and D, respectively) could comprehensively observe effects of all variables (Buelow and Suhr, 2009; Steingroever et al., 2013; Betz et al., 2018; Chiu et al., 2018). Some Sz-IGT studies have analyzed the four-deck format to compare the performance between both groups (Wilder et al., 1998; Ritter et al., 2004; Bark et al., 2005; Rodríguez-Sánchez et al., 2005; Shurman et al., 2005; Kester et al., 2006; Lee et al., 2007; Martino et al., 2007; Sevy et al., 2007; Kim et al., 2009, 2012, 2016; Wasserman et al., 2012; Hori et al., 2014; Brown et al., 2015; Matsuzawa et al., 2015; Zhang et al., 2015; Pedersen et al., 2017), but most have continued to adopt the EV viewpoint approach, ignoring the role of GLF in the decision-making process. Some studies have found that high winning frequency decks (B or D) are preferred by healthy control groups (Wilder et al., 1998; Dunn et al., 2006; Fernie, 2007; Chiu et al., 2012; Brown et al., 2015; Kim et al., 2016) when observing the four-deck pattern.

### Gain–Loss Frequency Viewpoint: IGT and “Prominent Deck B” Phenomenon

Observation of the four-deck format suggests that decision-making behavior on the IGT is affected not only by EV but also by GLF (Wilder et al., 1998; Chiu et al., 2012, 2018; Steingroever et al., 2013). Wilder et al. discovered that Sz cases favored decks B and D with high winning frequency (Wilder et al., 1998). Subsequently, Lin et al. reported that subjects preferred deck B with high winning frequency but negative EV and termed this as a “prominent deck B (PDB)” phenomenon (Lin et al., 2007), which contradicted the primary assumptions of the original IGT (Bechara et al., 1994).

The following IGT studies have suggested that GLF, rather than EV, was a critical factor influencing the decision-making behavior of the participants in IGT, which conflicts with primary IGT statements. Moreover, a series of relevant IGT studies have found that controls also preferred the disadvantageous deck B (Bark et al., 2005; Rodríguez-Sánchez et al., 2005; Fernie, 2007; Takano et al., 2010; Chiu et al., 2012; Steingroever et al., 2013). The PDB phenomenon of controls has gradually made an impact on the evaluation and development of IGT (Zhang et al., 2017; Chiu et al., 2018), including verifying IGT validity (Buelow and Suhr, 2009; Lin et al., 2013), constructing IGT decision-making models (Ahn et al., 2008; Lin et al., 2016), examining markers for sleep deprivation (Seeley et al., 2014, 2016), and examining the clinical application of IGT (Upton et al., 2012). Therefore, researchers have increasingly emphasized the association between the PDB phenomenon and IGT, suggesting that GLF plays an essential role in decision-making in healthy and neuropsychiatric individuals.

### Gain–Loss Frequency in Sz-IGT Studies

In Sz-IGT studies, the impact of GLF was initially discussed by Wilder et al. (1998). This study compared the number of four-deck choices between 12 Sz and 30 controls and found that both Sz and control picked more cards from decks B and D with high reward but low punishment frequency than decks A and C with low winning but high losing frequency. Wilder et al. (1998) considered that GLF might influence the decision-making behavior of Sz and control in IGT. Some subsequent Sz-IGT studies used (B+D) - (A+C), (B+D), and (A+C) as GLF measures to examine the difference between Sz and control groups (Rodríguez-Sánchez et al., 2005; Shurman et al., 2005; Kester et al., 2006; Brown et al., 2015), but no Sz-IGT study thoroughly discussed how GLF and PDB guide decision-making behavior in Sz cases. For instance, Sevy et al. conducted a review and experiment and found no significant difference across the net scores, deck A, deck B, deck C, and deck D between groups, but the selections of deck B were more than other decks within Sz and control groups (Sevy et al., 2007). Brown and colleagues conducted a brief meta-analysis on IGT and showed that Sz cases preferred deck B and control clearly preferred decks B and D. In the empirical phase, both Sz and control demonstrated more selections from deck B and deck D (Brown et al., 2015). A meta-analysis study investigated the decision-making performance of Sz cases across all IGT indices and showed that Sz preferred high winning frequency decks B and D (Betz et al., 2018). Based on this evidence, it is necessary to investigate the effect of GLF on the decision-making process in Sz cases.

Taken together, most Sz-IGT studies typically adopted a net score to represent individual decision-making abilities to detect schizophrenic behavior pattern from the EV viewpoint (Bechara et al., 1994; Ritter et al., 2004; Evans et al., 2005; Rodríguez-Sánchez et al., 2005; Shurman et al., 2005; Kester et al., 2006; Turnbull et al., 2006; Lee et al., 2007, 2009; Martino et al., 2007; Nakamura et al., 2007; Sevy et al., 2007; González-Blanch et al., 2008; Premkumar et al., 2008, 2015; Kim et al., 2009, 2012, 2016; Yip et al., 2009; Shirayama et al., 2010; Choi et al., 2011; Raffard et al., 2011; Struglia et al., 2011; Cella et al., 2012; Ayesa-Arriola et al., 2013; Brambilla et al., 2013; Fond et al., 2013; Hori et al., 2014; Nestor et al., 2014; Matsuzawa et al., 2015; Stratta et al., 2015; Zhang et al., 2015; Pedersen et al., 2017). The net score as a derivative measure drawing on the four decks A, B, C, and D might gloss over selections of each deck. This combination might not properly reveal that participants preferred the negative EV deck B (Horstmann et al., 2012). GLF as a potentially critical factor in decision-making behavior has not been investigated its role in Sz-IGT studies. Furthermore, most Sz-IGT studies also used WCST to examine the potential association between IGT and WCST. It was common to investigate the correlation between the net score (EV measure) and WCST; however, other IGT measures, such as decks B and D (GLF measures), were not frequently examined.

Accordingly, this study aimed to clarify the issues of inconsistency in Sz-IGT research based on EV and GLF viewpoints by using IGT, WCST, and clinical ratings and analyzing net score, four-deck format, and serial deck measures and their correlation with WCST. In order to assess whether Sz cases showed the PDB phenomenon, we compared the number of selections of decks under four-deck format, as well as D-A, D-B, D-C, C-B, C-A, and B-A. We predict that Sz cases will show the PDB phenomenon, namely, the number of deck B selection will be significantly higher than the other three decks. We argue that the disparity between decks A and B, which have exactly the same EV, supports the GLF viewpoint and violates the EV assumption.

## Materials and Methods

### Participants

Initially, 68 Sz or schizoaffective (SA) cases were recruited from Kaohsiung Municipal Siaogang Hospital and community mental health rehabilitation institutions. However, seven patients were unable to complete the whole procedure due to severe psychotic symptoms and eventually 61 chronic cases [mean age: 40.44 ± 11.23 (SD); 47.54% males] between the ages of 21 and 62 with Sz or SA disorder diagnosed by psychiatrists were included. From the community, 72 healthy adults, contacted via email, networking, and advertisements, were invited to participate. After the exclusion of 10 people who had a history of psychiatric or neurologic issues, this group consisted of 62 healthy subjects (mean age: 35.50 ± 15.10 (SD); 45.16% males) between the ages of 20 and 69, matched in age and gender. Exclusion criteria included the following: acute psychiatric instability, comorbid medical issues, brain injury, and meeting criteria for substance abuse or dependence. Healthy volunteers had no history of psychosis or neurological condition that would interfere with task performance. All participants received detailed information about the study procedures and provided their written informed consent. This study received ethical approval [No. KMUHIRB-SV(I)-20150075] from the Institutional Review Board of Kaohsiung Medical University Chung-Ho Memorial Hospital.

### General Procedure

All participants were provided with detailed information about procedures and voluntarily consented to receive assessments, including the IGT and WCST (Heaton et al., 1993) to evaluate affective decision-making and working memory, and problem-solving skills. By correlating these two measures, we sought to determine the relationship between decision-making behavior and the shifting flexibility in Sz cases. In Sz cases, the severity of overall psychiatric symptoms and the ability to self-care were assessed with the Positive and Negative Syndrome Scale (PANSS) and the Personal and Social Performance Scale (PSP). Healthy participants received a brief interview and were assessed with the exclusion criteria.

### Experimental Tasks

#### Iowa Gambling Task

The IGT version used in this study was corresponded closely to the structure of the original IGT (Bechara et al., 1994) (see Supplementary Table 2, Supplementary Figure 1). In our version, participants had a loan of NT$ 5,000 from the bank and were instantly informed of gained or lost money amount after each choice. They had 100 trials, which were not informed, to randomly choose from four decks and had to try their best to determine the winning strategy and maximize the money they gained. With regard to the IGT structure, each deck included 40 cards circulating with every 10 cards. Indeed, deck A and deck B were disadvantageous decks because of the negative expected value (EV) (NT*$*-250) based on every ten cards. If participants selected a card from deck A or B, they might receive a payoff of NT$100 or punishments ranging from NT*$*-150 to NT*$*-1250. Penalties of deck A were frequent and varied from NT*$*-150 to NT*$*-350, while punishments of deck B were infrequent, costing the participants NT$ 1250. In contrast, deck C and deck D were advantageous decks because of positive EV (NT$ +250). After selecting a card from deck C or D, participants might receive a reward of NT$ 50, or the penalties varied from NT*$*-25 to NT*$*-75. Penalties of deck C were frequent, and the amount ranged from NT*$*-25 to NT*$*-75, whereas punishments of deck D were infrequent and cost the participants NT $250. In terms of GLF of IGT, deck A and deck C had a similar gain–loss structure, which was 10 gains and 5 losses per 10 cards; for decks B and D, the structure was 10 gains and 1 loss per 10 cards (see Supplementary Table 2).

#### Wisconsin Card Sorting Test

The Wisconsin Card Sorting Test (WCST) was initially developed to assess the reasoning skills and the ability to shift cognitive strategies under environmental changes. What we administered was the computerized modification version from PEBL Version 0.14 (The PEBL Project, 2014). This test measures different cognitive functions involving executive functions, strategic planning, organized searching, set-shifting based on feedback information, goal-oriented behaviors, and modulation of impulsive responses (Heerey et al., 2008). WCST includes 4 stimulus cards and 128 response cards that differ in shape (cross, circle, triangle, or star), color (red, blue, yellow, or green), and number (one, two, three, or four). Participants were informed to correctly match response cards to one of the stimulus cards and were provided feedback after each selection. The matching rule will automatically switch to the next rule without informing subjects after 10 consecutive matchings. There is no time limit for this test, but the computer will automatically terminate when participants have completed 6 categories or when 128 cards have been all sorted. The primary outcome measure is perseverative errors (PE), and secondary outcome measures are total errors (TE), perseverative response (PR), non-perseverative errors (NPE), categories completed (CC), conceptual level (CL), and trials to complete the first category (TFC).

#### Positive and Negative Syndrome Scale

The Positive and Negative Syndrome Scale (PANSS) was used to assess the severity of psychiatric symptoms and social functions in Sz cases (Kay et al., 1987; Morosini et al., 2000). This study used the traditional Chinese version of PANSS and received authorization from Dr. Hwu and Dr. Huang, who standardized the traditional Chinese version in the National Taiwan University Hospital (Hwu et al., 1995). The scale covers positive symptoms (7 items), negative symptoms (7 items), general psychopathology scales (16 items), and supplementary items for the aggression risk profile (3 items), which accounts for a total of 33 items, for example, P1. Delusions: beliefs that are unfounded, unrealistic, and idiosyncratic (Kay et al., 1987). The severity of symptoms for each item is rated according to a 7-point scale (1 = absent; 7 = extreme). The reliability of the Chinese version of PANSS is within an acceptable range (0.76–0.78) (Hwu et al., 1995).

#### Personal and Social Performance Scale

Morosini et al. developed the Personal and Social Performance Scale (PSP) based on Social and Occupational Functioning Assessment (SOFAS) consisting of four main areas: (1) socially useful activities (e.g., housework and voluntary work), including work and study; (2) personal and social relationships (i.e., partner, family relationships, and friends); (3) self-care (i.e., personal hygiene and care for the appearance of an individual); and (4) disturbing and aggressive behavior (Morosini et al., 2000). Each area is rated on a 6-point scale from absent (no problems on this dimension) through mild, manifest, marked, and severe to very severe difficulties. PSP is highly correlated with SOFAS (*r* = 0.91) (Morosini et al., 2000). We used the traditional Chinese version of PSP that measures four dimensions: general function, interpersonal and social relations, ability to self-care, and interference and aggressive behavior (22 items in total) (Bai et al., 2014).

### Statistical Analysis

Regarding the demographic and clinical characteristics data, independent samples *t*-tests and chi-square tests were utilized to compare the matching level of gender, age, and education between two groups. The *t-*test was also used to examine the performance of WCST between Sz and control groups. For analyses of data from the IGT, independent samples *t*-tests and three separate ANOVAs were performed. Mauchly's test of sphericity was used to examine normality and homogeneity. If the results did not pass this test, we followed the Greenhouse–Geisser corrections. First, *t*-tests were performed to assess the group difference under a wide range of behavioral measures in IGT. Secondly, a two-way ANOVA using deck (four levels: A, B, C, and D) and group (Sz and control) as factors was performed to demonstrate the main effect of group and deck. After confirming the absence of a main effect of group, one-way ANOVAs were carried out to check for deck effects, respectively, in Sz and control. Scheffe *post-hoc* analyses were used to ascertain where differences in decks were present, verifying the PDB phenomenon. Third, we performed a three-way ANOVA with factors block (five levels: 20 trials per block), deck (four levels: A, B, C, and D), and group (Sz and control) to assess the group differences in learning performance on the four-deck format and all the behavioral outcome measures. Two-way ANOVAs, using block and deck as factors, and Scheffe *post-hoc* analyses were then carried out further to check block and deck effects separately in Sz and control groups. Finally, Pearson's correlation analyses were performed to explore possible relationships between IGT and measures of severity of symptom in the Sz group and between IGT and WCST within both groups. All statistical analyses were conducted using SPSS 19.0 software (IBM Corp, 2010).

## Results

### Demographic and Clinical Characteristics

A summary of demographic and clinical variables is shown in Table 1. Groups were well matched in gender and age; however, education level was significantly lower in the Sz group relative to the control group. The performance of the Sz group was significantly impaired in the WCST compared to the control group. Sz cases showed mild to moderate psychiatric dysfunction, personal and social dysfunction.

**Table 1:** Demographic and clinical characteristics of participants.

	**Sz** **(*N* = 61)**	**Control** **(*N* = 62)**	**Test statistic**	***p***	***d***
Gender (male: female)	29:32	28:34	χ^2^ = 0.70	0.86	
Age	40.44 (11.23)	35.50 (15.10)	*t =* 2.06	0.42	−0.37
Education	12.41 (2.32)	15.34 (2.22)	*t =* −7.16^**^	<0.001^***^	1.29
WCST-TC	50.92 (18.36)	63.15 (16.42)	*t =* −3.89^***^	<0.001^***^	0.70
WCST-TE	72.49 (26.20)	45.03 (31.42)	*t =* 5.27^***^	<0.001^***^	0.90
WCST-PR	48.23 (35.20)	26.21 (27.96)	*t =* 3.84^***^	<0.001^***^	0.65
WCST-PE	39.72 (25.87)	22.66 (21.36)	*t =* 3.99^***^	<0.001^***^	0.66
WCST-NE	32.77 (23.56)	22.37 (20.64)	*t =* 2.61^*^	0.02^*^	0.43
WCST-CL	31.00 (25.20)	51.37 (23.38)	*t =* −4.65^***^	<0.001^***^	0.84
WCST-CC	1.90 (2.17)	4.05 (2.35)	*t =* −5.26^***^	<0.001^***^	0.95
WCST-TFC	66.46 (54.39)	38.21 (43.70)	*t =* 3.17^**^	0.002^**^	−0.57
PANSS-T	67.49 (14.91)	–	–		
PANSS-P	15.62 (5.30)	–	–		
PANSS-N	16.82 (4.74)	–	–		
PANSS-G	29.95 (6.54)	–	–		
PSP	69.70 (8.99)	–	–		

*Sz, Schizophrenia; WCST, Wisconsin Cards Sorting Test; PANSS, The Positive and Negative Syndrome Scale; TC, Total corrects; TE, Total errors; PR, Perseverative response; PE, Perseverative errors; NE, Non-perseverative errors; CC, Categories completed; CL, Conceptual level; TFC, Trials to first category; T, Total score of PANSS; P, Positive symptoms; N, Negative symptoms; G, general psychopathology scales; PSP, Personal and Social Performance Scale.*

* *p < 0.05,*

** *p < 0.01, and*

*** *p < 0.001*.

### IGT Results: Behavioral Measures

The data comparing a wide range of IGT behavioral measures between Sz and control groups under *t*-test in Table 2 illustrated that there was no significant difference between the two groups. Namely, the Sz group demonstrated a similar decision-making level relative to the control group. Two-way ANOVA on all measures across five blocks (20 trials as one block) also revealed no main effects of group and no interaction effect of group and block but the main effect of block on (C+D) - (A+B) [*F*_(3, 292)_ = 2.62, *p* = 0.46, ${\eta_{p}}^{2}$ = 0.02], (B+D)-(A+C) [*F*_(3, 404)_ = 3.23, *p* = 0.02, ${\eta_{p}}^{2}$ = 0.03], D-A [*F*_(3, 362)_ = 2.99, *p* = 0.04, ${\eta_{p}}^{2}$ = 0.04], C-A [*F*_(3, 413)_ = 4.83, *p* = 0.002, ${\eta_{p}}^{2}$ = 0.04], B-A [*F*_(3, 377)_ = 2.89, *p* = 0.03, ${\eta_{p}}^{2}$ = 0.02]. Indeed, the Sz group showed a comparable level of decision-making performance relative to the control group, but a differentiation across five blocks within groups was observed, suggesting that the two groups might have distinct reward learning processes.

**Table 2:** IGT performance of Sz and control.

	**Sz**	**Control**	***t***	***p***	***d***
Total earned money	4631.15 (596.77)	4425.81 (621.13)	1.87	0.06	−0.34
(C+D)-(A+B)	−2.89 (16.51)	−5.00 (18.54)	0.67	0.51	−0.12
C+D	48.56 (8.26)	47.50 (9.27)	0.67	0.51	−0.12
A+B	51.44 (8.26)	52.50 (9.27)	−0.67	0.51	0.12
(B+D)-(A+C)	9.90 (15.67)	6.26 (14.55)	1.34	0.18	−0.24
B+D	54.95 (7.84)	53.13 (7.28)	1.34	0.18	−0.24
A+C	45.05 (7.84)	46.87 (7.28)	−1.34	0.18	0.24
D-A	3.51 (10.60)	0.63 (11.07)	1.47	0.14	−0.27
D-B	−6.66 (13.56)	−8.00 (13.63)	0.55	0.59	−0.10
D-C	−0.26 (11.20)	−2.37 (10.26)	1.09	0.28	−0.20
C-A	3.77 (8.18)	3.00 (10.84)	0.44	0.66	−0.08
C-B	−6.39 (12.18)	−5.63 (12.46)	−0.35	0.73	0.08
B-A	10.16 (10.58)	8.63 (11.49)	0.77	0.44	−0.18

*Sz, Schizophrenia.*

### IGT Results: Prominent Deck B Phenomenon

**Figure 1:**
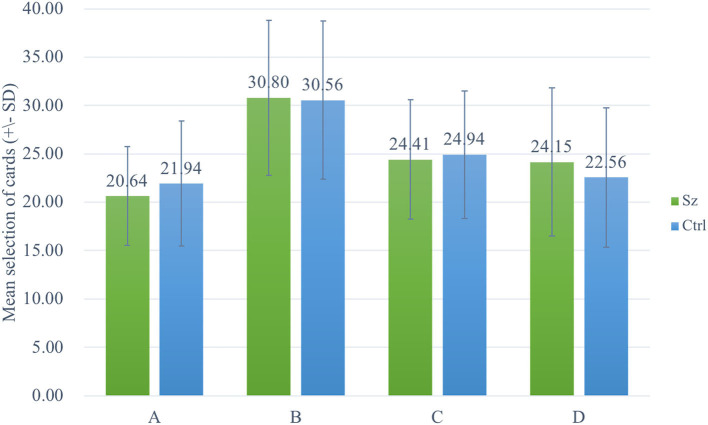
Selective patterns of Sz and control. Green bars represent the Sz group, whereas blue bars represent the control group. Both groups selected more cards from deck B than from the other decks.

**Figure 2:**
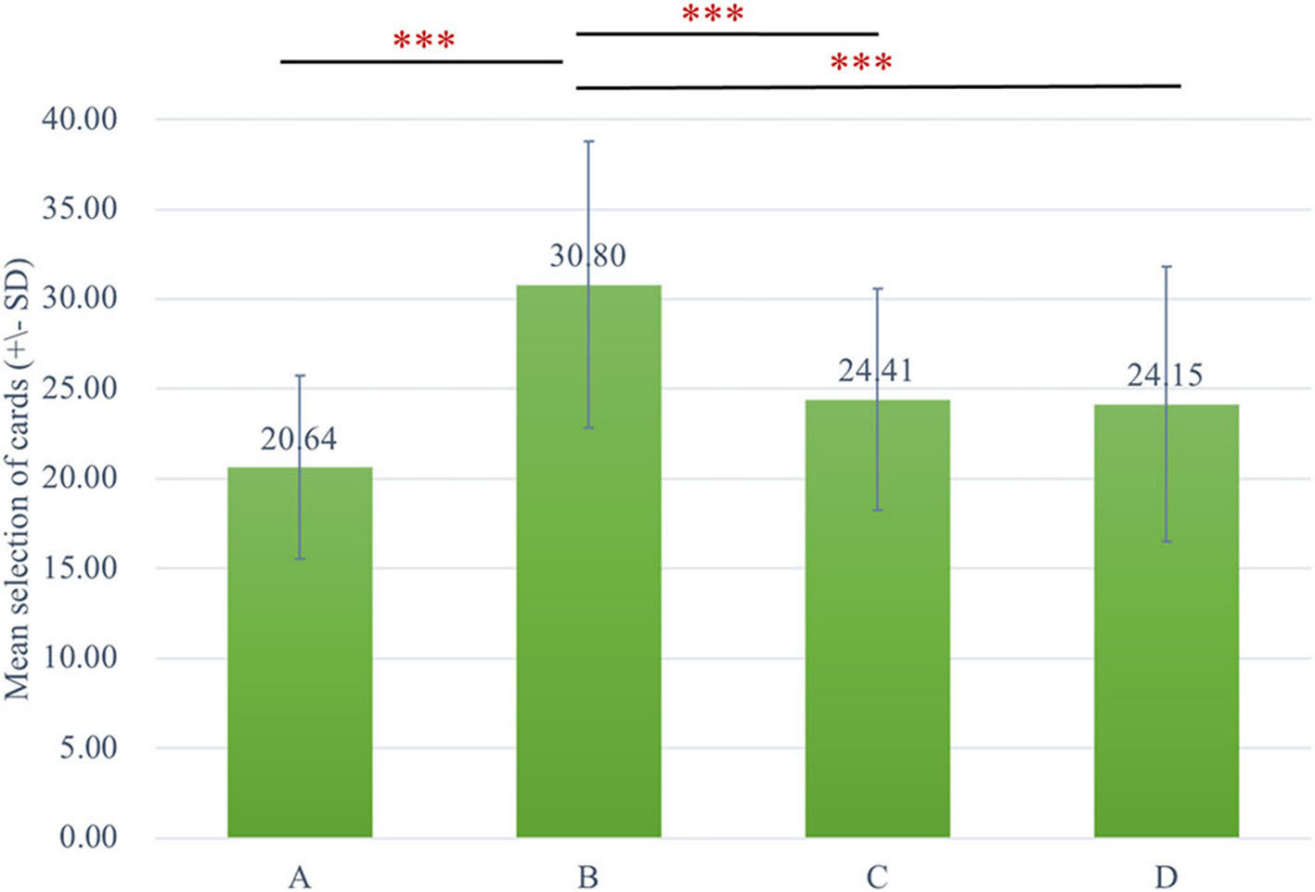
The selective pattern of Sz. Green bars represent the four-deck format of the Sz group, and the number of selections from deck B is significantly larger than the other three decks. ***p < 0.001.

**Figure 3:**
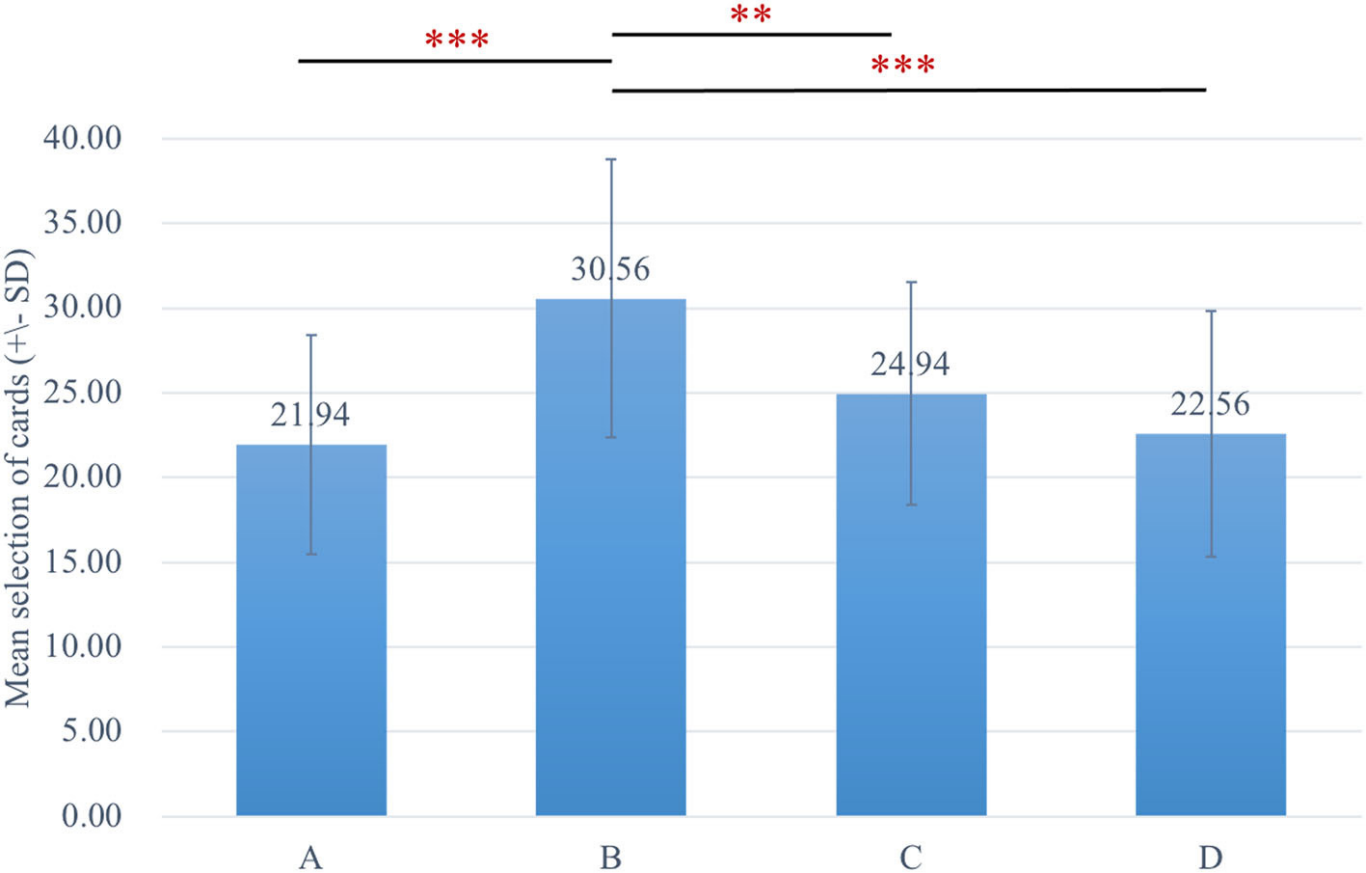
The selective pattern of control. Blue bars represent the four-deck format of the control group, and the number of selections from deck B is significantly larger than the other three decks. **p < 0.01, ***p < 0.001.

The two-way ANOVA using the four-deck format in Figure 1 revealed no significant main effect of group and interaction of group and deck, while the main effect of deck [*F*_(3, 326)_ = 30.72, *p* < 0.001, ${\eta_{p}}^{2}$ = 0.20] was statistically significant with a small effect size. As there was no group difference in four-deck selective patterns, one-way ANOVAs were conducted to elucidate the factors in IGT, respectively, for Sz and control groups as shown in Table 3, indicating the main effect of the deck in both groups with small effect size [Sz: *F*_(3, 154)_ = 17.56, *p* < 0.001, ${\eta_{p}}^{2}$ = 0.23; control: *F*_(3, 167)_ = 14.03, *p* < 0.001, ${\eta_{p}}^{2}$ = 0.19]. From Scheffe *post-hoc* analysis of four decks in Figures 2, 3, it appears that both the Sz and control groups both preferred high-gain frequency deck B (Sz: B>A^***^, B>C^***^, and B>D^***^; control: B>A^***^, B>C^**^, and B>D^***^), suggesting that both groups had PDB phenomenon.

**Table 3:** Four-deck format patterns in Sz and control.

	**A**	**B**	**C**	**D**	***F***	***p***	**$\eta_{p}^{2}$**	***Post-hoc***
Sz	20.64 (5.11)	30.80 (8.00)	24.41 (6.17)	24.15 (7.66)	17.56	<0.001^***^	0.23	B>A^***^, B>C^***^, B>D^***^
Control	21.94 (6.47)	30.56 (8.19)	24.94 (6.59)	22.56 (7.23)	14.03	<0.001^***^	0.19	B>A^***^, B>C^**^, B>D^***^

*The distribution did not meet the spherical hypothesis, and the Greenhouse–Geisser correction was adopted. The Scheffe method was used in the post-hoc analysis.*

** *p < 0.01,*

*** *p < 0.001*.

**Figure 4:**
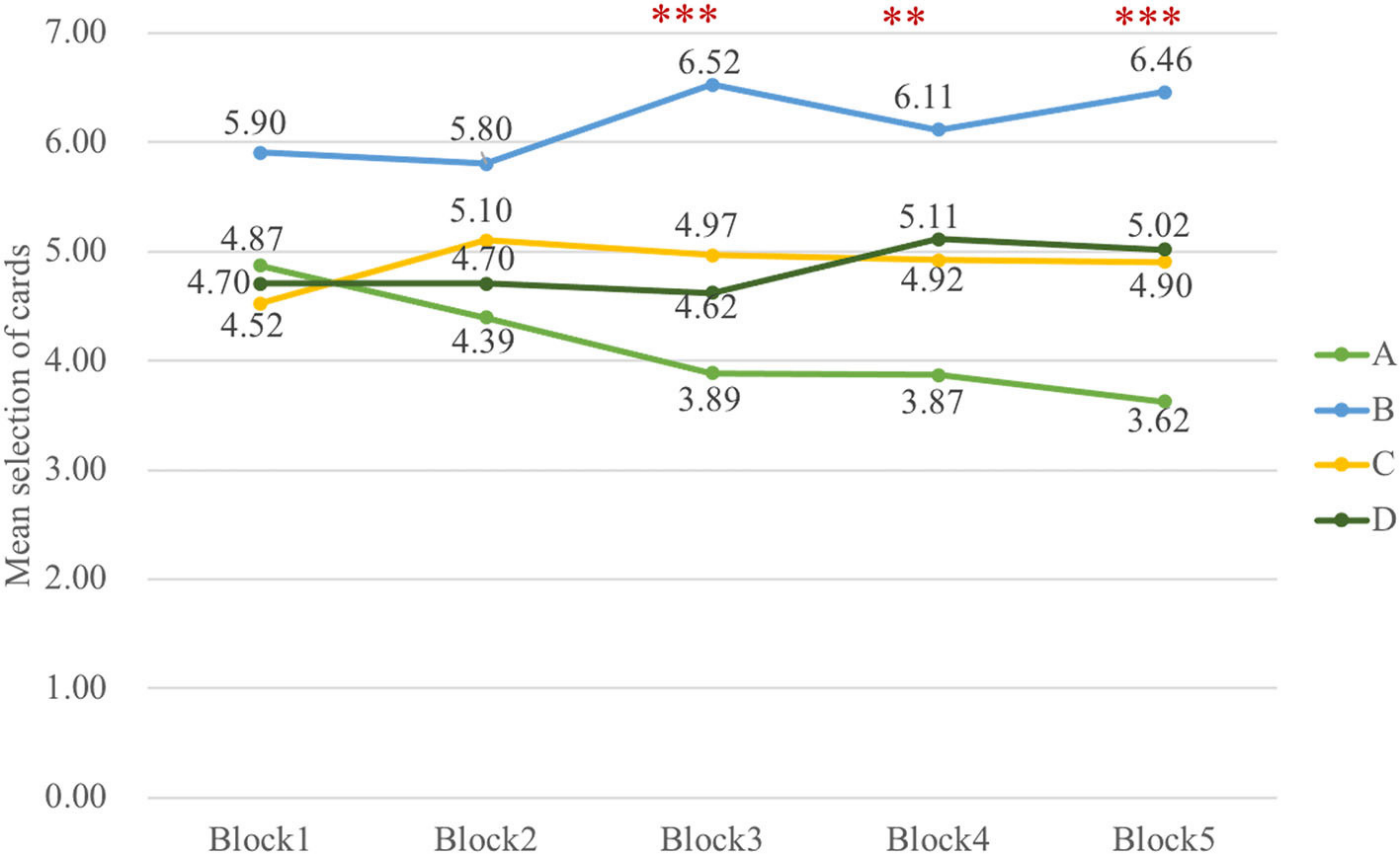
The selective pattern of Sz in five blocks. The number of cards from deck B is significantly larger than deck A over block 3 [*F*_(3, 493)_ = 7.18, *p* < 0.001], block 4 [*F*_(3, 493)_ = 4.92, *p* = 0.002], and block 5 [*F*_(3, 493)_ = 7.87, *p* < 0.001] in Sz. **p < 0.01, ***p < 0.001.

**Figure 5:**
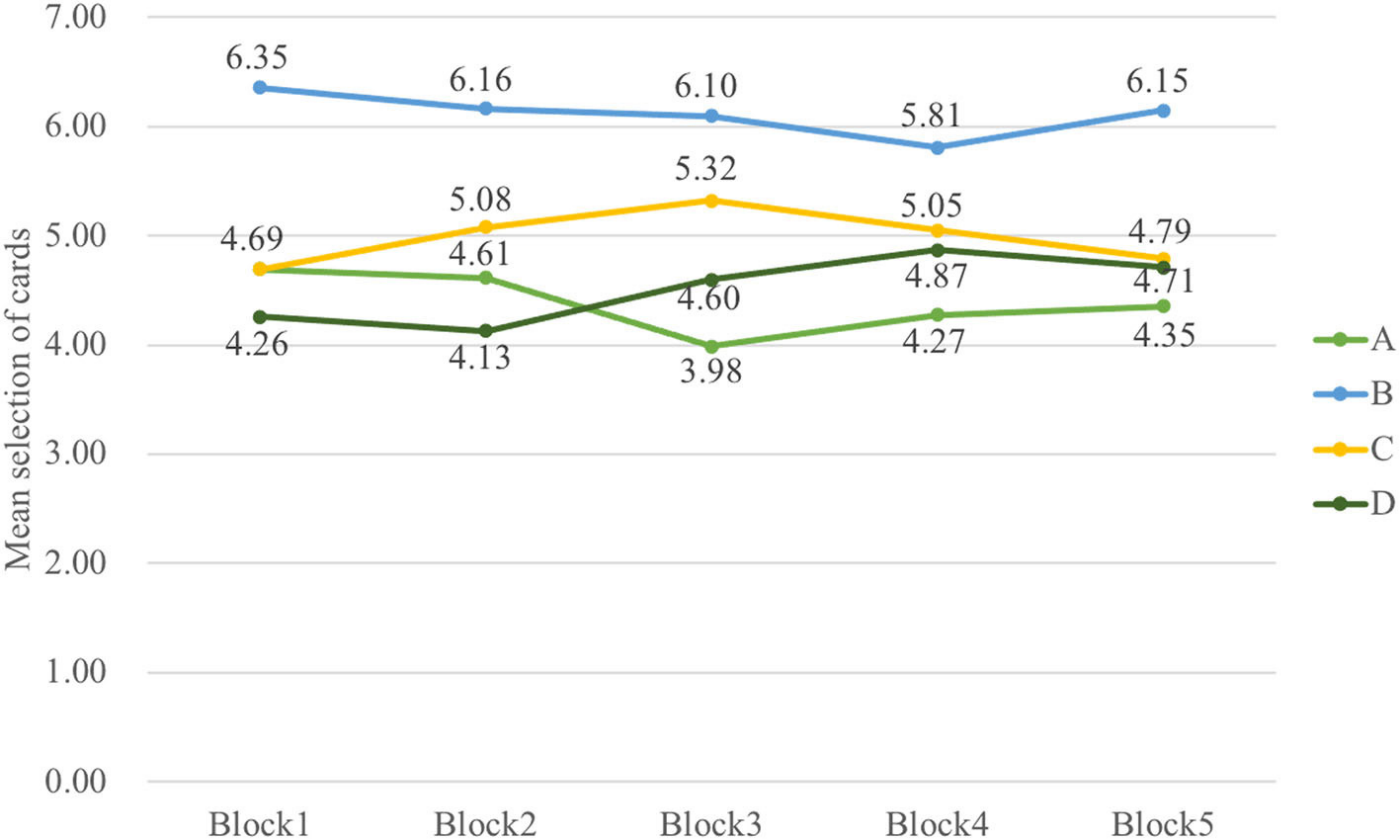
The selective pattern of control in five blocks. No significant results are shown from the learning curve in control. The control group did not learn to choose fewer cards from disadvantageous deck B, but they tended to choose fewer cards from deck A over the last three blocks.

Indeed, the data in a three-way ANOVA analyzing block-by-block learning process indicated no main effect of group and block, but it indicated that the main effect of deck [*F*_(3, 326)_ = 30.72, *p* < 0.001, ${\eta_{p}}^{2}$ = 0.20] and interaction of block and deck [*F*_(8, 985)_ = 2.51, *p* = 0.01, ${\eta_{p}}^{2}$ = 0.02] were significant. To examine the selective patterns of both groups in learning progress, two-way ANOVAs were carried out in Sz and control groups, respectively. The outcomes illustrated the main effect of deck [*F*_(3, 154)_ = 17.53, *p* < 0.001, ${\eta_{p}}^{2}$ = 0.23] and interaction of deck and block [*F*_(6, 339)_ = 2.26, *p* = 0.04, ${\eta_{p}}^{2}$ = 0.04] in Sz cases and only a main effect of deck [*F*_(3, 167)_ = 14.05, *p* < 0.001, ${\eta_{p}}^{2}$ = 0.19] in control participants. The results in Figure 4 also revealed that Sz cases had more selections from deck B than other decks over block 3 [*F*_(3, 493)_ = 7.18, *p* < 0.001, ${\eta_{p}}^{2}$ = 0.04], block 4 [*F*_(3, 493)_ = 4.92, *p* = 0.002, ${\eta_{p}}^{2}$ = 0.03], and block 5 [*F*_(3, 493)_ = 7.87, *p* < 0.001, ${\eta_{p}}^{2}$ = 0.05] (see Figure 5). PDB became more dominant in the late learning phase in Sz cases; however, this pattern was not seen in control participants. Two-way ANOVAs were also performed to examine the group and block effects across all the behavioral outcome measures, but no group differences were found.

### Correlation Analysis

Results from analyses of correlations between IGT and WCST performance metrics in Sz are presented in Table 4. Positive significant correlations were observed between deck D and TE (*r* = 0.33, *p* = 0.008), CC (*r* = 0.44, *p* < 0.001), and CL (*r* = 0.37, *p* = 0.01), while TFC were inversely correlated with deck D (*r* = −0.32, *p* = 0.01). Deck B was positively correlated with NE (*r* = 0.30, *p* = 0.02). Positive correlations between net score and TE (*r* = 0.27, *p* = 0.03), NE (*r* = 0.33, *p* = 0.01), CC (*r* = 0.35, *p* = 0.01), and CL (*r* = 0.33, *p* = 0.01) were also showed. No correlations were found between measures of the severity of symptom and IGT. Reward-based decision-making performance in IGT was related to the executive function in WCST rather than clinical symptoms in Sz cases.

**Table 4:** Results of correlation of IGT and WCST in Sz.

	**A**	**B**	**C**	**D**	**(C+D)-(A+B)**	**(B+D)-(A+C)**
TE	−0.10	−0.22	−0.05	0.33^**^	0.27^*^	0.10
PR	−0.04	−0.02	−0.08	0.12	0.05	0.09
PE	−0.05	−0.03	−0.07	0.12	0.06	0.09
NE	−0.07	0.30^*^	0.16	0.23	0.33^**^	−0.08
CC	−0.21	−0.23	−0.08	0.44^***^	0.35^**^	0.20
CL	−0.19	−0.23	−0.02	0.37^**^	0.33^**^	0.14
TFC	0.15	0.12	0.12	−0.32^*^	−0.21	−0.19

*TE, Total errors; PR, Perseverative response; PE, Perseverative errors; NE, Non-perseverative errors; CC, Categories completed; CL, Conceptual level; TFC, Trials to first category.*

* *p < 0.05,*

** *p < 0.01,*

*** *p < 0.001*.

## Discussion

This is the first study to examine whether IGT has feasible outcome measures for identifying risky decision-making behavior and pinpointing the role of PDB in IGT for Sz cases. We found no suitable primary measures in IGT to identify decision-making process deficits of Sz cases relative to the control group. However, the Sz group demonstrated the decision-making pattern with substantially more choices from the disadvantageous deck B than other decks, particularly in the later phase of the learning processing. Thus, individuals with Sz showed a robust and stable PDB phenomenon with evidence in our study, suggesting decision-making behavior under risk in the Sz cohort was highly guided by GLF, and this effect got enhanced in the learning process.

Our results were supported by a review and empirical studies, which did not find significant differences across the net score, deck A, deck B, deck C, and deck D (Wilder et al., 1998; Sevy et al., 2007; Glick et al., 2021). A categorical score (categorical score = 1 if Σ net scores for trials 1–60 or trials 61–100 ≥ 0, and = 0 if Σ net scores for trials 1–60 or trials 61–100 <0) was defined in Sevy et al. study, and it showed a significant difference between Sz and control groups (Sevy et al., 2007); however, Sz preferring deck B was also observed, which was not reported by authors. Two Sz-IGT meta-analysis studies showed significant differences in EV and GLF measures (Betz et al., 2018; Li et al., 2019). Li et al. found a moderate-sized effect of the net score for Sz compared to healthy individuals in a meta-analytic approach (Li et al., 2019); nevertheless, this study did not examine the potential heterogeneous sources and the effects of four decks. In contrast, Betz et al. performed a meta-analysis study for all IGT outcome measures in Sz and showed significant effects of the net score (block 2 to 5) and significant effect sizes across decks A, B, and D (Betz et al., 2018). Unfortunately, the present study did not replicate these results. One potential reason is that some particular studies derived the results of meta-analyses. For example, in deck analyses, findings of meta-regression were driven by Zhang et al. (2015) and the results were no longer significant once this study was omitted (Betz et al., 2018). Additionally, the above meta-analysis studies only included studies published after 2000, and one crucial clinical trial (Wilder et al., 1998) was omitted, which may affect the results of meta-analyses. Furthermore, Lee et al. (2020) recollected over 900 IGT-related studies and found out 86 studies of them reported data with the four-deck format and their observation demonstrated over half of the studies (58/86, 67.44%) presented the PDB phenomenon in healthy/control groups. Namely, the PDB phenomenon and the preference of participants for disadvantageous deck B have profoundly affected the explanation of IGT performance. Accordingly, it is worth noting the presence of the PDB phenomenon not only in the healthy/control group but also in the patient group (see also Supplementary Figure 3).

### PDB Phenomenon

A noticeable preference for the deck B with frequent gains and rare large losses, over the deck of moderate losses (deck A), is consistent with several previously reported findings in Sz cases (Ritter et al., 2004; Bark et al., 2005; Rodríguez-Sánchez et al., 2005; Shurman et al., 2005; Kester et al., 2006; Lee et al., 2007; Martino et al., 2007; Kim et al., 2009, 2012, 2016; Wasserman et al., 2012; Hori et al., 2014; Brown et al., 2015; Matsuzawa et al., 2015; Zhang et al., 2015; Pedersen et al., 2017). Even if the net score of Sz was significantly lower than control in some studies, Sz group still presenting a robust PDB phenomenon was well in line with the general pattern of responses of Sz cases in the analysis of previous studies (Kim et al., 2009, 2016; Wasserman et al., 2012; Matsuzawa et al., 2015; Zhang et al., 2015) (see also Supplementary Figure 3).

Notably, previous IGT studies usually deciphered the PDB phenomenon in accordance with the definition of the study of Bechara et al. (1994) and tended to report that patients appeared to choose more cards from disadvantageous decks fitting the assumption of EV viewpoint but overlooked the significant disparity between decks B and A with the same negative EV. The first Sz-IGT study observed that Sz cases made more choices from deck B than deck A (Wilder et al., 1998), in line with other Sz-IGT studies (Brown et al., 2015), supporting the GLF viewpoint. Sevy et al. did not find the group difference among a wide range of IGT behavioral measures but observed that the Sz group preferred deck B rather than deck A (Sevy et al., 2007). A newly published study displaying the four-deck format also revealed the group difference on the net score and also showed that Sz cases had the tendency of selecting high gaining frequency decks (Saperia et al., 2019). A meta-analysis study provided the evidence that net scores between Sz and control groups were significantly different; nevertheless, the Sz and control groups both preferred to choose deck B as well (Betz et al., 2018) (see also Supplementary Figure 3).

These outcomes indicate that net score as a derivative behavioral measure consisting of four decks glosses over the choices of each deck. This might partly explain why the PDB phenomenon was not initially discovered. In particular, the integration of net score could not properly reveal that participants preferred deck B with negative EV (Horstmann et al., 2012). Some studies suggest that the four-deck format should be measured and presented along with other IGT measures (Horstmann et al., 2012; Zhang et al., 2015). Simply observing net score overlooks the PDB phenomenon, which is completely contradictory to the EV viewpoint proposing that VMPFC-impaired cases cannot acknowledge the concept of EV and select more cards from disadvantageous decks with negative EV, while the control group can progressively learn the concept of EV and prefer advantageous decks with positive EV. Importantly, Pan et al. (2010) applied simplified IGT, (i.e., AACC and BBDD versions), and found that the Sz group choosing equally from decks B and D was insensitive to EV, violating the basic hypothesis of IGT. This suggests that GLF may be the dominant factor affecting the decision-making process of Sz cases (Pan et al., 2010).

The remarkable PDB phenomenon of Sz may stem from insensitivity to large monetary penalties (Heerey et al., 2008; Brown et al., 2013). Brown et al. designed an experiment based on the framing effect and found that Sz had risker behavior under a negative frame than controls under uncertain scenarios due to insensitivity to losses (Brown et al., 2013). Brown et al. assessed Sz cases with IGT and the Balloon Analog Risk Task (BART) and argued that Sz cases are more likely to have a reinforcement learning deficit, specifically involving the integration of frequencies and magnitudes of rewards and punishments in the trial-by-trial estimation of EV (Brown et al., 2015). A recent study also claimed that Sz cases demonstrated intact lose–shift behavior, but significantly reduced win–stay rates compared to healthy controls in IGT (Saperia et al., 2019). Failure to learn a successful strategy in the IGT may be linked to deficits in reversal learning in Sz (Fellows and Farah, 2004; Dunn et al., 2006). Researchers using distinct decision-making tasks suggested that Sz cases have impaired reversal learning ability, leading to value-based decision-making and reinforcement learning dysfunction (Fellows and Farah, 2004; Waltz and Gold, 2007; Sterzer et al., 2019).

### Unstable Factors in IGT

Extensive analyses on a wide range of IGT outcome measures used in Sz-IGT studies show that EV measures are not suitable for discriminating the Sz group from the control group. This is consistent with several previously reported findings in relation to Sz (Wilder et al., 1998; Bark et al., 2005; Evans et al., 2005; Rodríguez-Sánchez et al., 2005; Turnbull et al., 2006; Martino et al., 2007; Sevy et al., 2007; González-Blanch et al., 2008; Shirayama et al., 2010; Choi et al., 2011; Carvalho et al., 2012; Ayesa-Arriola et al., 2013; Premkumar et al., 2015; Pedersen et al., 2017). Likewise, GLF measures cannot be discriminative behavioral measures, consistent with three studies that found no significant difference in GLF measures (Rodríguez-Sánchez et al., 2005; Kester et al., 2006; Carvalho et al., 2012; Brown et al., 2015). Comparisons among several assessments specific to VMPFC dysfunction frontotemporal dementia cases revealed that IGT was not capable of detecting VMPFC impairment relative to other assessments (Bertoux et al., 2013).

Surprisingly, the same level of decision-making capability was shown in both Sz and control groups even if multiple lines of evidence suggested OFC dysfunction in cases with Sz, including evidence of reduced volumes (Larquet et al., 2010; Kanahara et al., 2013), task-evoked hypoactivity (Quintana et al., 2003), and impairments in reversal learning (Waltz and Gold, 2007). In addition to the Sz exhibiting the notable PDB, control showing a robust PDB is also observed (see Figure 1), which has been proved to be a stable phenomenon in a series of studies concerning original, modified, and clinical versions of IGT (Lin et al., 2007, 2009, 2012, 2013; Chiu et al., 2012; Fernie and Tunney, 2013). IGT performance of control had a considerable variation (Chiu et al., 2012), and another review paper also supported this line of thought (Steingroever et al., 2013). This might reveal a gap in IGT performance and an unstable selective pattern of control groups existing in Sz-IGT studies [detail review and analyses please see Xu (2018)]. The inconsistent observation of the IGT selective pattern of control is likely due to the heterogeneity of healthy participants with various demographic variables (Dunn et al., 2006; Chiu et al., 2012; Steingroever et al., 2013). Gender makes significant difference in deck selections, as women make more choices from deck B than from deck D compared to men (Overman, 2004; Singh et al., 2020). High-education and low-education groups perform significantly differently in terms of net score in the last two blocks (Evans et al., 2004). The older subjects preferred to choose more cards from disadvantageous deck A (Carvalho et al., 2012).

### Correlations

It is reasonable to observe the correlation between WCST and IGT (deck B, deck D, net score) as some neuroimaging studies have reexamined that IGT is related to VMPFC and DLPFC (Fellows and Farah, 2004; Maia and McClelland, 2004; Lin et al., 2008; Li et al., 2009). Moreover, previous Sz-IGT studies also reported that the net score was related to WCST (Lee et al., 2009; Yip et al., 2009; Brambilla et al., 2013; Nestor et al., 2014), but only one study found deck D negatively correlated with perseverative errors (Shurman et al., 2005). The correlation between four decks and the WCST has been neglected in previous studies, which explains why few of them have found a relationship between decks with high winning frequency and the WCST. Every correct feedback as a reward imposes an impact on a selection made by the participants in assisting them to learn the rule and completing one category. The frequency of “right” feedback on WCST and the frequency of gains in IGT significantly affect the learning process.

On the other hand, we did not observe meaningful correlations between performance in IGT and the severity of positive symptoms in line with most published studies (Evans et al., 2005; Lee et al., 2007, 2009; Martino et al., 2007; Premkumar et al., 2008; Kim et al., 2009, 2012; Fond et al., 2013; Hori et al., 2014; Stratta et al., 2015; Pedersen et al., 2017), and the absence of correlation for negative symptoms is also supported by previous studies (Ritter et al., 2004; Evans et al., 2005; Kester et al., 2006; Lee et al., 2007; Martino et al., 2007; Premkumar et al., 2008; Kim et al., 2009, 2012; Struglia et al., 2011; Fond et al., 2013; Hori et al., 2014; Brown et al., 2015; Stratta et al., 2015; Pedersen et al., 2017) in Sz cases, which may be due to the generally low severity of symptoms of the Sz cases in our sample. Sz cases with delusion proneness selected more advantageously on the IGT relative to those scoring lower without delusion proneness (Runyon and Buelow, 2019). Participants in our study are chronic patients with relieved positive symptoms (i.e., delusion), and their decision-making behavior may not be guided by positive symptoms at this stage. An alternative explanation may be that the risky decision-making ability in IGT is not necessarily correlated with clinical symptoms.

### Limitations and Future Directions

The first limitation of this study concerns the potential confounding effects that come from antipsychotic medication and doses of usage on IGT performance. Group difference on IGT performance was found in Sz cases under different drug therapies (Beninger et al., 2003). Betz and colleagues performed a meta-analysis study and showed that a higher dose of antipsychotic medication was associated with decreased net scores during early blocks and diagnosis was associated with a lower net score and moderated immediate gains in Sz (Betz et al., 2018). As our study included both Sz and SA disorders, different diagnoses likely affected decision-making performance in IGT. Second, the education level of Sz and control were not well-matched, which might be a potential confounding factor. Some evidence suggested that participants with lower education levels showed better performance on the IGT than those with higher education levels (Evans et al., 2004). Third, as for the study design, we did not counterbalance the order of IGT and WCST.

An adequate assessment of these issues is only possible in the context of a controlled clinical trial with randomized assignment to identical diagnoses, identical drugs, and well-matched education. Future studies should also consider making reasonable classifications, for example, different types of antipsychotic medicines, if the characteristics of the participants are diverse. The IGT version utilized in this study was the original version (1994) developed by Bechara's group instead of a clinical version: PAR IGT (2007) which has been claimed to examine reward-based decision-making deficit across 13 different neuropsychological disorders (Bechara, 2007). PAR IGT showed that participants made fewer deck B selections during the early trials (1–40) and later trials (41–100) relative to the original IGT (Buelow and Barnhart, 2017). However, the selective patterns of PAR and original IGT in the study by Buelow were similar to our findings. Lin et al. recruited 72 healthy participants to investigate whether deck B was preferred in PAR IGT, and it turned out that PDB was stable in this clinical version (Lin et al., 2013). Further investigation regarding PDB in Sz is required to verify our findings in PAR IGT.

### Conclusions

Both Sz and control groups exhibited the PDB phenomenon, and the net score, a combination of four decks, based on the EV viewpoint does indeed obscure the PDB phenomenon of Sz and control, making it difficult to observe the PDB phenomenon. GLF potentially imposes a considerable effect on two populations. IGT as a research tool enables researchers to observe the risky decision-making behaviors of participants under the guidance of several factors; however, it is not qualified as a clinical assessment to evaluate the decision-making functioning only judging based on the EV makers, since the PDB violates the EV hypothesis. Hence, future investigations should prioritize empirical experiments on clinical IGT and to confirm these observations reflected in Sz cases. In clinical scenarios, we strongly advise against evaluating and diagnosing decision-making dysfunction purely on the basis of net score on the clinical IGT.

## Data Availability Statement

The raw data supporting the conclusions of this article will be made available by the authors, without undue reservation.

## Ethics Statement

The studies involving human participants were reviewed and approved by the Institutional Review Board of Kaohsiung Medical University Chung-Ho Memorial Hospital [No. KMUHIRB-SV(I)-20150075]. The patients/participants provided their written informed consent to participate in this study.

## Author Contributions

MX, W-KL, C-HK, Y-CC, and C-HL contributed to the conceptual innovation, literature review, experimental design, statistical analysis, and drafting of the study. More than two authors were involved in intense and frequent discussions whenever disagreements appeared. MX initially conducted the literature review, experimental design, data analysis, and data collection and finally drafted the initial study. W-KL concentrated on checking data, and provided constructive suggestions and thoughtful ideas in the draft of the study. C-HK screened eligible participants and provided suggestions regarding recruitment institutes and clinical assessments. Y-CC was constructed the whole study, provided partial research studies, gave critical suggestions, and revised the study. C-HL worked on constructing the structure of this study, data analysis, interpretation, provided vital ideas in discussion, development and refining of the study. All authors contributed to the article and approved the submitted version.

## Conflict of Interest

The authors declare that the research was conducted in the absence of any commercial or financial relationships that could be construed as a potential conflict of interest.

## Publisher's Note

All claims expressed in this article are solely those of the authors and do not necessarily represent those of their affiliated organizations, or those of the publisher, the editors and the reviewers. Any product that may be evaluated in this article, or claim that may be made by its manufacturer, is not guaranteed or endorsed by the publisher.
