# Supplementary material for: The Prominent Deck B Phenomenon in Schizophrenia: An Empirical Study on Iowa Gambling Task
Source: Front Psychol. 2021 Sep 3;12:619855. doi: 10.3389/fpsyg.2021.619855 (PMC8446202; doi:10.3389/fpsyg.2021.619855)
Supplement: Supplementary file 1 [file Data_Sheet_1.docx]

Supplementary Material

# Supplementary Tables

**Table S1. Results of IGT performance in 38 studies**

| **Authors** | **Subjects** ^a^ | | | | | | | | | | | | **Assessments** ^b^ | **Indicators of IGT** ^c^ | **Results** |
| --- | --- | --- | --- | --- | --- | --- | --- | --- | --- | --- | --- | --- | --- | --- | --- |
| **Studies showing difference in IGT performance between two groups** | | | | | | | | | | | | | | | |
| **Beninger et al. (2003)** |  | 18 Sz (typicals) | | | 18 Sz (atypicals) | | | | | | 18 Ctrl | | IGT  WCST  DMQ  MMSE  BPRS | 1. C+D | Atypicals > Typicals or controls (C+D) |
|  | Age | 45.6 ±1.4 | | | 42.1±2.3 | | | | | | 45.2±2.8 | |  |  |  |
|  | Gen. | 67% male | | | 67% male | | | | | | 67% male | |  |  |  |
|  | Edu. | - | | | - | | | | | | - | |  |  |  |
| **Ritter et al. (2004)** |  | 20 Sz | | | | 15 Ctrl | | | | | | | IGT  NART  WCST  BPRS  SANS | 1. A, B, C, D 2. (C+D)-(A+B) 3. C+D 4. A+B 5. MG 6. ML 7. NG | Sz < Ctrl [(C+D)-(A+B)]  Sz < Ctrl (C+D)  Sz > Ctrl (A+B)  Sz < Ctrl (NG)  Sz > Ctrl (ML) |
|  | Age | 48.5±6.0 | | | | 47.1±10.2 | | | | | | |  |  |  |
|  | Gen. | 100% male | | | | 100% male | | | | | | |  |  |  |
|  | Edu. | 13.8±1.70 | | | | 13.3±1.45 | | | | | | |  |  |  |
| **Shurman et al. (2005)** |  | 39 Sz | | | | 10 Ctrl | | | | | | | IGT  WCST  DMST  SANS  SAPS  PANSS | 1. (C+D)-(A+B) 2. MAM 3. A, B, C, D | Sz < Ctrl [(C+D)-(A+B)]  Sz < Ctrl (MAM)  Sz > Ctrl (A)  Sz > Ctrl (B)  Sz < Ctrl (C) |
|  | Age | 33.5±10.1 | | | | 32.1±4.5 | | | | | | |  |  |  |
|  | Gen. | 72% male | | | | 50% male | | | | | | |  |  |  |
|  | Edu. | 13.4±1.2 | | | | 15.5±2.4 | | | | | | |  |  |  |
| **Kester et al. (2006)** |  | 15 Sz | | | | 25 Ctrl | | | | | | | IGT  WRAT-III  WCST  BPRS  SANS  PAS  CGAS | 1. (C+D)-(A+B) 2. A, B, C, D 3. EVL 4. (B+D)-(A+C) | Sz < Ctrl [total (C+D)-(A+B), (C+D)-(A+B) in block 4 and 5]  Sz > Ctrl (A)  Sz < Ctrl (D) |
|  | Age | 15.9±2.7 | | | | 17.1±1.8 | | | | | | |  |  |  |
|  | Gen. | 60% male | | | | 56% male | | | | | | |  |  |  |
|  | Edu. | - | | | | - | | | | | | |  |  |  |
| **Lee et al. (2007)** |  | 23 Sz | | | | 28 Ctrl | | | | | | | IGT  GDT  SRLT  WCST  PANSS(P, N, G) | 1. (C+D)-(A+B) 2. A, B, C, D | Sz < Ctrl [(C+D)-(A+B)]  Sz > Ctrl (B)  Sz < Ctrl (D) |
|  | Age | 27.6±5.5 | | | | 26.9±3.6 | | | | | | |  |  |  |
|  | Gen. | 70% male | | | | 54% male | | | | | | |  |  |  |
|  | Edu. | 14.1±2.0 | | | | 14.3±1.5 | | | | | | |  |  |  |
| **Nakumura et al. (2008)** |  | 24 Sz | | | | 25 Ctrl | | | | | | | IGT  TMT  WCST  PANSS | 1. (C+D)-(A+B) 2. ML 3. MG | Sz < Ctrl [(C+D)-(A+B)]  Sz > Ctrl (ML)  Sz < Ctrl (MG) |
|  | Age | 39.1±10.3 | | | | 41.1±9.1 | | | | | | |  |  |  |
|  | Gen. | 100% male | | | | 76% male | | | | | | |  |  |  |
|  | Edu. | - | | | | - | | | | | | |  |  |  |
| **Premkumar et al. (2008)** |  | 75 Sz | | | | 25 Ctrl | | | | | | | IGT  PANSS | 1. (C+D)-(A+B) 2. EVL | Sz < Ctrl [(C+D)-(A+B) from block 2 to 5]  Sz < Ctrl ($E_{j}(t)$ of EVL) |
|  | Age | 37.9±9.5 | | | | 35.4±11.9 | | | | | | |  |  |  |
|  | Gen. | 73.3% male | | | | 64% male | | | | | | |  |  |  |
|  | Edu. | - | | | | - | | | | | | |  |  |  |
| **Kim et al. (2009)** |  | 52 Sz | | | | 55 Ctrl | | | | | | | IGT  WAIS-R  WCST  PANSS | 1. (C+D)-(A+B) 2. A, B, C, D | Sz < Ctrl [(C+D)-(A+B)]  Sz > Ctrl (B)  Sz < Ctrl (D) |
|  | Age | 30.6±5.9 | | | | 28.8±7.5 | | | | | | |  |  |  |
|  | Gen. | 57.7% male | | | | 52.7% male | | | | | | |  |  |  |
|  | Edu. | 12.7±9.3 | | | | 14.8±1.9 | | | | | | |  |  |  |
| **Lee et al. (2009)** |  | 39 Sz | | | | 33 Ctrl | | | | | | | IGT  FAIT  WCST  An abbreviated form of WAIS-R  PANSS | 1. (C+D)-(A+B) | Sz < Ctrl [(C+D)-(A+B)] |
|  | Age | 32.4±7.2 | | | | 29.0±8.9 | | | | | | |  |  |  |
|  | Gen. | 48.7% male | | | | 42.4% male | | | | | | |  |  |  |
|  | Edu. | 12.7±1.9 | | | | 14.7±1.8 | | | | | | |  |  |  |
| **Yip et al. (2009)** |  | 10 Ctrl (non-smokers) | 9 Ctrl (smokers) | | | 10 Sz (non-smokers) | | | | | | 32 Sz (smokers) | IGT  WCST  BDI  BPRS  PANSS | 1. (C+D)-(A+B) 2. A+B | Sz < Ctrl [(C+D)-(A+B)]  Sz < Ctrl (A+B in block 5)  Sz (female non-smoker) < Sz (female smoker) [(C+D)-(A+B)]  Atypicals < Typicals [(C+D)-(A+B)] |
|  | Age | 36.7±13.7 | 38.0±8.8 | | | 39.3±9.6 | | | | | | 41.0±6.3 |  |  |  |
|  | Gen. | 83.3% male | 77.8% male | | | 50.0% male | | | | | | 76.7% male |  |  |  |
|  | Edu. | 16.1±2.6 | 13.2±2.6 | | | 13.6±1.2 | | | | | | 11.7±2.2 |  |  |  |
| **Struglia et al. (2011)** |  | 40 Sz  (30 atypical and 10 typical) | | | | 20 Ctrl | | | | | | | IGT  PANSS | 1. (C+D)-(A+B) 2. MAM 3. A+B 4. C+D | Sz < Ctrl [(C+D)-(A+B)]  Sz > Ctrl (A+B)  Sz < Ctrl (C+D) |
|  | Age | 41.35±10.29 | | | | 42.35±11.75 | | | | | | |  |  |  |
|  | Gen. | 67.5% male | | | | 55% male | | | | | | |  |  |  |
|  | Edu. | 10.35±3.06 | | | | 12.25±3.35 | | | | | | |  |  |  |
| **Raffard et al. (2011)** |  | 64 Ctrl | | 27 Sz (high insight) | | | | | | 37 Sz (low insight) | | | IGT  PANSS  f-NART | 1. (C+D)-(A+B) | Sz (both hight and low insight groups) < Ctrl [(C+D)-(A+B) in block 3, 4, 5] |
|  | Age | 33.6±11 | | 35.4±10.6 | | | | | | 33.5±11.9 | | |  |  |  |
|  | Gen. | 42% male | | 61% male | | | | | | 83% male | | |  |  |  |
|  | Edu. | 12.2±2.8 | | 10.9±2.4 | | | | | | 10.4±2.3 | | |  |  |  |
| **Wasserman et al. (2012)** |  | 26 Ctrl | | 19 Sz (Typicals) | | | | | | 53 Sz (Atypical) | | | IGT  PCL  PANSS  MMSE  DMQ | 1. C+D 2. A, B, C, D 3. DMQ for IGT   (analyze the first 80 trials) | Ctrl > Typicals and Atypicals (C+D) |
|  | Age | 48.2±2.8 | | 43.3±3.3 | | | | | | 39.9±1.7 | | |  |  |  |
|  | Gen. | 57.7% male | | 78.9% male | | | | | | 81.1% male | | |  |  |  |
|  | Edu. | 15.1±0.4 | | 12.2±0.4 | | | | | | 12.2±0.4 | | |  |  |  |
| **Kim et al. (2012)** |  | 30 Sz | | | | 33 Ctrl | | | | | | | IGT  VGT  SGT  WCST  PANSS | 1. (C+D)-(A+B) 2. A, B, C, D | Sz < Ctrl [(C+D)-(A+B)]  Sz > Ctrl (B)  Sz < Ctrl (D) |
|  | Age | 63.3% male | | | | 48.5% male | | | | | | |  |  |  |
|  | Gen. | 29.2±5.7 | | | | 27.8±3.0 | | | | | | |  |  |  |
|  | Edu. | 14.0±1.9 | | | | 14.8±1.4 | | | | | | |  |  |  |
| **Cella et al. (2012)** |  | 25 Sz | | | | 24 Ctrl | | | | | | | IGT  SAR PSYRATS  SANS | 1. (C+D)-(A+B) 2. SAR 3. EVL | Sz < Ctrl [(C+D)-(A+B) in block 3, 4 and 5] |
|  | Age | 9.24±10.54 | | | | 35±10.51 | | | | | | |  |  |  |
|  | Gen. | 62.5% male | | | | 44% male | | | | | | |  |  |  |
|  | Edu. | 14.3±2.12 | | | | 15.38±1.76 | | | | | | |  |  |  |
| **Fond et al. (2013)** |  | 63 Sz | | | | 67 Ctrl | | | | | | | IGT  GDT  TMT  HCST  PANSS  BDI | 1. (C+D)-(A+B) | Sz < Ctrl [(C+D)-(A+B)] |
|  | Age | 34.6±11.53 | | | | 34.57±12.36 | | | | | | |  |  |  |
|  | Gen. | 25.4% female | | | | 55.2% female | | | | | | |  |  |  |
|  | Edu. | 10.65±2.35 | | | | 12.13±2.77 | | | | | | |  |  |  |
| **Brambilla et al. (2013)** |  | 70 Sz | | | | 140 Ctrl | | | | | | | IGT  BPRS  WAIS-R  WCST. | 1. (C+D)-(A+B) 2. EVL | Sz < Ctrl [(C+D)-(A+B)]  Sz < Ctrl (EVL) |
|  | Age | 44.2±10.9 | | | | 43.9±11.2 | | | | | | |  |  |  |
|  | Gen. | - (incorrect information) | | | | 50.7% male | | | | | | |  |  |  |
|  | Edu. | - | | | | - | | | | | | |  |  |  |
| **Hori et al. (2014)** |  | 86 Sz | | | | | | | 51 Ctrl | | | | IGT in Japanese  JART  BDNF  PANSS (P, N, G, T) | 1. MAM 2. A, B, C, D 3. (C+D)-(A+B) | Sz < Ctrl (MAM)  Sz > Ctrl (B)  Sz < Ctrl (C)  Sz < Ctrl [(C+D)-(A+B)] |
|  | Age | 35.1±12.1 | | | | | | | 36.7±9.9 | | | |  |  |  |
|  | Gen. | 50.0% male | | | | | | | 49.0% male | | | |  |  |  |
|  | Edu. | 12.7±2.7 | | | | | | | 13.4±2.2 | | | |  |  |  |
| **Nestor et al. (2014)** |  | 65 Sz | | | | | | | 65 Ctrl | | | | IGT  WAIS  WMS  WCST  SANS  SAPS | 1. (C+D)-(A+B) 2. NG | Sz < Ctrl (NG)  Sz < Ctrl [(C+D)-(A+B) from block 2 to 5] |
|  | Age | 42.19±10 | | | | | | | 41.59±8.49 | | | |  |  |  |
|  | Gen. | - | | | | | | | - | | | |  |  |  |
|  | Edu. | 13.32±1.93 | | | | | | | 14.90±1.96 | | | |  |  |  |
| **Matsuzawa et al. (2015)** |  | 61 Sz  (37 residual type, 24 paranoid type) | | | | 50 Ctrl | | | | | | | IGT in Japanese  BPRS  SANS  DIEPSS  WAIS-R | 1. A, B, C, D 2. (C+D) 3. (A+B) 4. (C+D)-(A+B) 5. (C+D)-(A+B) (41-100); [(41-100)-(1-20)] 6. (C+D)/(A+B+C+D) | Sz < Ctrl (C+D)-(A+B)([41-100])  Sz < Ctrl (C+D)-(A+B)([41-100]-[1-20]). |
|  | Age | 34.3±8.3 | | | | 31.9±7.8 | | | | | | |  |  |  |
|  | Gen. | 54.1% male | | | | 66% male | | | | | | |  |  |  |
|  | Edu. | 13.8±2.2 | | | | 15.4±3.1 | | | | | | |  |  |  |
| **Brown et al. (2015)** |  | 59 Sz | | | | 43 Ctrl | | | | | | | IGT  BART  BPRS  SANS  RBANS WASI  LSN  DSST | 1. (A+B)/100 2. (C+D)/100 3. (B+D)/100 4. Percentage of A, B, C, D 5. MAM | Sz < Ctrl [(C+D)/100 in block 4 and 5]  Sz > Ctrl (A)  Sz < Ctrl (D) |
|  | Age | 42.2±11.8 | | | | 41.1±11.8 | | | | | | |  |  |  |
|  | Gen. | 71.2% male | | | | 60.5% male | | | | | | |  |  |  |
|  | Edu. | 12.6±2.7 | | | | 14.8±2.2 | | | | | | |  |  |  |
| **Stratta et al. (2015)** |  | 30 Sz | | | | 32 Ctrl | | | | | | | IGT  SLOF  PANSS | 1. (C+D)-(A+B) 2. MAM 3. (C+D)-(A+B) (under ambiguity; under risk) | Sz < Ctrl [total (C+D)-(A+B)]  Sz < Ctrl [(C+D)-(A+B) in block 4, 5 and under risk] |
|  | Age | 18-65 | | | | matched | | | | | | |  |  |  |
|  | Gen. | 60% male | | | | 50% male | | | | | | |  |  |  |
|  | Edu. | matched | | | | matched | | | | | | |  |  |  |
| **Zhang et al.(2015)** |  | 46 Sz | | | | 80 Ctrl | | | | | | | IGT  GDT  WCST  TMT  DST  PANSS | 1. (C+D)-(A+B) 2. A, B, C, D | Sz < Ctrl [total (C+D)-(A+B)]  Sz < Ctrl [(C+D)-(A+B) in block 4, 5]  Sz > Ctrl (B)  Sz < Ctrl (D) |
|  | Age | 19.9±3.76 | | | | 19.2±2.96 | | | | | | |  |  |  |
|  | Gen. | 80.4% male | | | | 83.75% male | | | | | | |  |  |  |
|  | Edu. | 10.5±1.8 | | | | 10.6±1.4 | | | | | | |  |  |  |
|  | IQ | 106.5±16.0 | | | | 108±14.3 | | | | | | |  |  |  |
| **Kim et al. (2016)** |  | 39 Sz  (30 paranoid, 9 undifferentiated) | | | | | 31 Ctrl | | | | | | IGT  PANSS | 1. (C+D)-(A+B) 2. A, B, C, D 3. PVL | Sz < Ctrl [total (C+D)-(A+B)]  Sz < Ctrl [(C+D)-(A+B) in block 4, 5]  Sz < Ctrl (D) |
|  | Age | 38.85±9.52 | | | | | 38.32±9.12 | | | | | |  |  |  |
|  | Gen. | 46.15% male | | | | | 45.16% male | | | | | |  |  |  |
|  | Edu. | 13.03±2.12 | | | | | 13.96±2.40 | | | | | |  |  |  |
|  | IQ | 96.44±12.51 | | | | | 111.84±10.50 | | | | | |  |  |  |
| **Studies showing no difference in IGT performance between two groups** | | | | | | | | | | | | | | | |
| **Wilder et al. (1998)** |  | 12 Sz | | | | 30 Ctrl | | | | | | | IGT  CVLT  LNSP  WAIS-R  WRAT-R reading test | 1. A, B, C, D 2. MAM | Sz = Ctrl (A, B, C, D, MAM)  B, D > A, C (Both in Sz and Ctrl) |
|  | Age | 33±6 | | | | 30±10 | | | | | | |  |  |  |
|  | Gen. | 91% male | | | | 41% male | | | | | | |  |  |  |
|  | Edu. | 13.1±2.5 | | | | 15.2±2.4 | | | | | | |  |  |  |
| **Cavallaro et al. (2003)** |  | 110 Sz | | | | 56 Ctrl | | | | | | | IGT  WCST  TH | 1. A+B | Sz = Ctrl (A+B) |
|  | Age | 33 ± 9.5 | | | | 31.2 ± 6.0 | | | | | | |  |  |  |
|  | Gen. | 60% male | | | | 39.2% male | | | | | | |  |  |  |
|  | Edu. | 12.7 ± 2.4 | | | | 13.5 ± 2.8 | | | | | | |  |  |  |
| **Evans et al. (2005)** |  | 19 Sz | | | | 19 Ctrl | | | | | | | IGT  SAR  WASI  WCST  COWAT  SANS  SAPS | 1. (C+D)-(A+B) 2. SAR | Sz = Ctrl [(C+D)-(A+B), SAR] |
|  | Age | 38.3±10.4 | | | | matched | | | | | | |  |  |  |
|  | Gen. | - | | | | - | | | | | | |  |  |  |
|  | Edu. | 12.8 ± 1.77 | | | | matched | | | | | | |  |  |  |
| **Rodríguez-Sánchez et al. (2005)** |  | 80 Sz | | | | 22 Ctrl | | | | | | | IGT  BG  FAS  TMT  SANS  SAPS | 1. (C+D)-(A+B) 2. A+B 3. B+D 4. A+C 5. A, B, C, D | Sz = Ctrl [(C+D)-(A+B), A+B, A, B, C, D]  B+D < A+C (Both in Sz and Ctrl) |
|  | Age | 25.7±6.7 | | | | 26.1±6.5 | | | | | | |  |  |  |
|  | Gen. | 68.8% male | | | | 54.5% male | | | | | | |  |  |  |
|  | Edu. | 11.3±2.7 | | | | 12.1±2.2 | | | | | | |  |  |  |
| **Bark et al. (2005)** |  | 8 Sz (catatonic) | | 19 Sz (paranoid) | | | | | | 26 Ctrl | | | IGT  OAT  GNG  VWM  WCST  DAT  SPM  MWT-B  GAS  PANSS | 1. A, B, C, D | Sz = Ctrl |
|  | Age | 36.8±10.5 | | 38.5±14 | | | | | | 30±9 | | |  |  |  |
|  | Gen. | 37.5% male | | 65% male | | | | | | 46% male | | |  |  |  |
|  | Edu. | 9.2±1.1 | | 9.8±1.4 | | | | | | - | | |  |  |  |
| **Turnbull et al. (2006)** |  | 21 Sz | | | | 21 Ctrl | | | | | | | IGT  WAIS  COWAT  WCST  BADS  SAPS  SANS | 1. (C+D)-(A+B) 2. PGNB | Sz = Ctrl [(C+D)-(A+B)] |
|  | Age | 38.3±10.4 | | | | 36.14±8.9 | | | | | | |  |  |  |
|  | Gen. | 61.9% male | | | | 61.9% male | | | | | | |  |  |  |
|  | Edu. | 12.6±1.0 | | | | 12.6±1.0 | | | | | | |  |  |  |
| **Martino et al. (2007)** |  | 21 Sz | | | | 15 Ctrl | | | | | | | IGT  WASI  MBS  SANS  PANSS | 1. A, B, C, D 2. (C+D)-(A+B) 3. MAM | Sz = Ctrl [(C+D)-(A+B), MAM]  Sz > Ctrl (A)  Sz < Ctrl (D) |
|  | Age | 32.7±9.0 | | | | 35.0±10.9 | | | | | | |  |  |  |
|  | Gen. | 58% male | | | | 40% male | | | | | | |  |  |  |
|  | Edu. | 10.4±2.2 | | | | 10.6±1.8 | | | | | | |  |  |  |
| **Sevy et al. (2007)** |  | 27 Sz  (14 concurrent cannabis use disorders) | | | | | 20 Ctrl | | | | | | IGT  WRAT-3  CPT-IP  WAIS-R  Digit Span  CVLT  COWAT  TMT | 1. NG 2. A, B, C, D 3. (C+D)-(A+B) 4. Categorical scores 5. EVL | Sz = Ctrl [NG, A, B, C, D, (C+D)-(A+B), EVL] |
|  | Age | 30±9 | | | | | 33±10 | | | | | |  |  |  |
|  | Gen. | 63% male | | | | | 60% male | | | | | |  |  |  |
|  | Edu. | 12±2 | | | | | 15±2 | | | | | |  |  |  |
| **González-Blanch et al. (2008)** |  | 70 Sz | | | | 21 Ctrl | | | | | | | IGT  OAT | 1. (C+D)-(A+B) | Sz = Ctrl [(C+D)-(A+B)] |
|  | Age | 26.4±6.7 | | | | 25.9±6.3 | | | | | | |  |  |  |
|  | Gen. | 61.4% male | | | | 57.1% male | | | | | | |  |  |  |
|  | Edu. | 10.1±2.7 | | | | 11.9±2.8 | | | | | | |  |  |  |
| **Shirayama et al. (2010)** |  | 19 Sz | | | | 18 Ctrl | | | | | | | IGT  VFT  WCST  Stroop  TMT  DSDT  BPRS  SANS  DIEPSS | 1. (C+D)-(A+B) | Sz = Ctrl [(C+D)-(A+B)] |
|  | Age | 30.5±5.6 | | | | 31.4±8.4 | | | | | | |  |  |  |
|  | Gen. | 63.2% male | | | | 77.8% male | | | | | | |  |  |  |
|  | Edu. | 13.7±1.8 | | | | 15.4±3.2 | | | | | | |  |  |  |
| **Choi et al. (2011)** |  | 25 Sz | | | | 23 Ctrl | | | | | | | IGT  SAPS  SANS | 1. (C+D)-(A+B) 2. MG 3. ML 4. NG | Sz = Ctrl [(C+D)-(A+B), MG, ML, NG] |
|  | Age | 44.7 ± 9.7 | | | | 42.2 ± 10.1 | | | | | | |  |  |  |
|  | Gen. | 100% male | | | | 100% male | | | | | | |  |  |  |
|  | Edu. | 13.2 ± 1.8 | | | | 14.8 ± 2.0 | | | | | | |  |  |  |
| **Ayesa-Arriola et al. (2013)** |  | 79 Sz | | | | | | | | | | 41 Ctrl | IGT  SAPS  SANS  CDSS  SARS  BAS  RAVLT  RCFT  GP  CPT  TMT-B  BG  Digit symbol  FAS | 1. (C+D)-(A+B) | Sz = Ctrl [(C+D)-(A+B)] |
|  |  | 28 haloperidol | 23 olanzapine | | | | | 28 risperidone | | | |  |  |  |  |
|  | Age | 26.9±6.9 | 27.4±7.5 | | | | | 27.9±9.2 | | | | 28.1±8.0 |  |  |  |
|  | Gen. | 71.4% male | 56.5% male | | | | | 57.1% male | | | | 51.2% male |  |  |  |
|  | Edu. | 10.2±2.8 | 11.6±3.3 | | | | | 10.5±2.8 | | | | 11.2±2.4 |  |  |  |
| **Premkumar et al. (2015)** |  | 25 Sz  (22 paranoid, 2 not specified, 1 schizoaffective) | | | | 25 Ctrl | | | | | | | IGT  PANSS | 1. (C+D)-(A+B) 2. EVL | Sz = Ctrl [(C+D)-(A+B), EVL] |
|  | Age | 35.6±8.1 | | | | 33.7±12.6 | | | | | | |  |  |  |
|  | Gen. | 68% male | | | | 68% male | | | | | | |  |  |  |
|  | Edu. | 13.9±3.1 | | | | 15.1±2.5 | | | | | | |  |  |  |
| **Pedersen et al. (2017)** |  | 38 Sz | | | | 38 Ctrl | | | | | | | IGT  GDT  PANSS | 1. (C+D)-(A+B) 2. A, B, C, D 3. MAM | Sz = Ctrl [(C+D)-(A+B), A, B, C, MAM]  Sz < Ctrl (D) |
|  | Age | 40.1±14.0 | | | | 40.0±13.8 | | | | | | |  |  |  |
|  | Gen. | 57.89% male | | | | 57.89%male | | | | | | |  |  |  |
|  | Edu. | 11.0±1.7 | | | | 11.6±1.6 | | | | | | |  |  |  |

^a^: Gen. = gender; Edu. = education; Sz = schizophrenia; Ctrl = control.^b^: WCST = Wisconsin Card Sorting Test; DMQ= Declarative Memory Questionnaire; MMSE = Mini Mental State Exam; BPRS = Brief Psychiatric Rating Scale; NART = North American Reading Test; SANS = Scale for the Assessment of Negative Symptoms; SAPS = Scale for the Assessment of Positive Symptoms; DMST = delayed match to sample task; PANSS = Positive and Negative Syndrome Scale (Subscale: P = Positive, N = Negative, G = General Psychopathology); WRAT = Wide Range Achievement Test; PAS = Premorbid Adjustment Scale; CGAS = Children’s Global Assessment Scale Score; GDT = Game of Dice Task; SRLT = Simple Reversal Learning Test; TMT = Trail Making Test; WAIS-R = Wechsler Adult Intelligence Scale-Revised; FAIT = Facial Affect Identification Test; BDI = Beck Depression Inventory; f-NART = French Version of the National Adult Reading Test ; PCL = Weather Prediction Probabilistic Classification Learning; DMQ = declarative memory questionnaires for IGT and PCL; VGT = Variant gambling task; SGT = Shuffled Gambling Task; HCST = Hayling Sentence completion task; UMT = Updating Memory Task; SAR = subjective awareness rating; PSYRATS = Psychotic Symptom Rating Scale; JART = Japanese Adult Reading Test; BDNF = Brain-derived neurotrophic factor measurement; DIEPSS = Drug Induced Extrapyramidal Symptoms Scale; BART = Balloon Analog Risk Task; RBANS = Repeatable Battery for the assessment of neuropsychological status; LNS = Letter Number Sequencing Test; DSST = Digit Symbol Substitution Test; SLOF = Specific Level of Function Scale; DST = Digit Span Test; CVLT = California Verbal Learning Test; LNSP = Letter Number Span; WRAT-R = Wide Range Achievement Test-Revised; TH = Tower of Hanoi; COWAT = Controlled Oral Word Association Test; BG = WAIS backward digits; FAS = fluency test; CPT = Continuous Performance Test; OAT = Object Alternation Task; GNG = Go-No Go Task; VWM = verbal working memory; DAT = divided attention task; SPM = Standard Progressive Matrices; MWT-B = Multiple Vocabulary Test-B; GAS = Global Assessment Scale; BADS = Behavioral Assessment of the Dysexecutive Syndrome; MBS = Memory Battery of Signoret; LSHS = Launay-Slade Hallucination Scale; PDI = Peter et al. Delusions Inventory; VFT = Verbal Fluency Test; DSDT = Digit span distraction test; CDSS = Calgary Depression Scale for Schizophrenia; SARS = Simpson-Angus Rating Scale; BAS = Barnes Akathisia Scale; RAVLT = Rey Auditory Verbal Learning Test; RCFT = Rey Complex Figure Test; GP = Grooved pegboard. ^c^: NG= net gain, MG= money gained, ML= money lost, EVL= Expectancy valence model, PVL= Prospect valence model, MAM= mean amount of money, SAR= subjective awareness ratings, PGNB = Mean number of selections of previously-good-now-bad decks.

**Table S2. Structure of original IGT**

| IGT | A | B | C | D |
| --- | --- | --- | --- | --- |
| 1 | 100 | 100 | 50 | 50 |
| 2 | 100 | 100 | 50 | 50 |
| 3 | 100, -150 | 100 | 50, -50 | 50 |
| 4 | 100 | 100 | 50 | 50 |
| 5 | 100, -300 | 100 | 50, -50 | 50 |
| 6 | 100 | 100 | 50 | 50 |
| 7 | 100, -200 | 100 | 50, -50 | 50 |
| 8 | 100 | 100 | 50 | 50 |
| 9 | 100, -250 | 100, -1250 | 50, -50 | 50 |
| 10 | 100, -350 | 100 | 50, -50 | 50, -250 |
| Expected value  (10 cards) | -250 | -250 | +250 | +250 |
| Gain-loss frequency  (10 cards) | 10 gains  5 losses | 10 gains  1 loss | 10 gains  5 losses | 10 gains  1 loss |

10 cards constitute a loop in IGT structure, and this table only presents the first 10 cards.

# Supplementary Figures


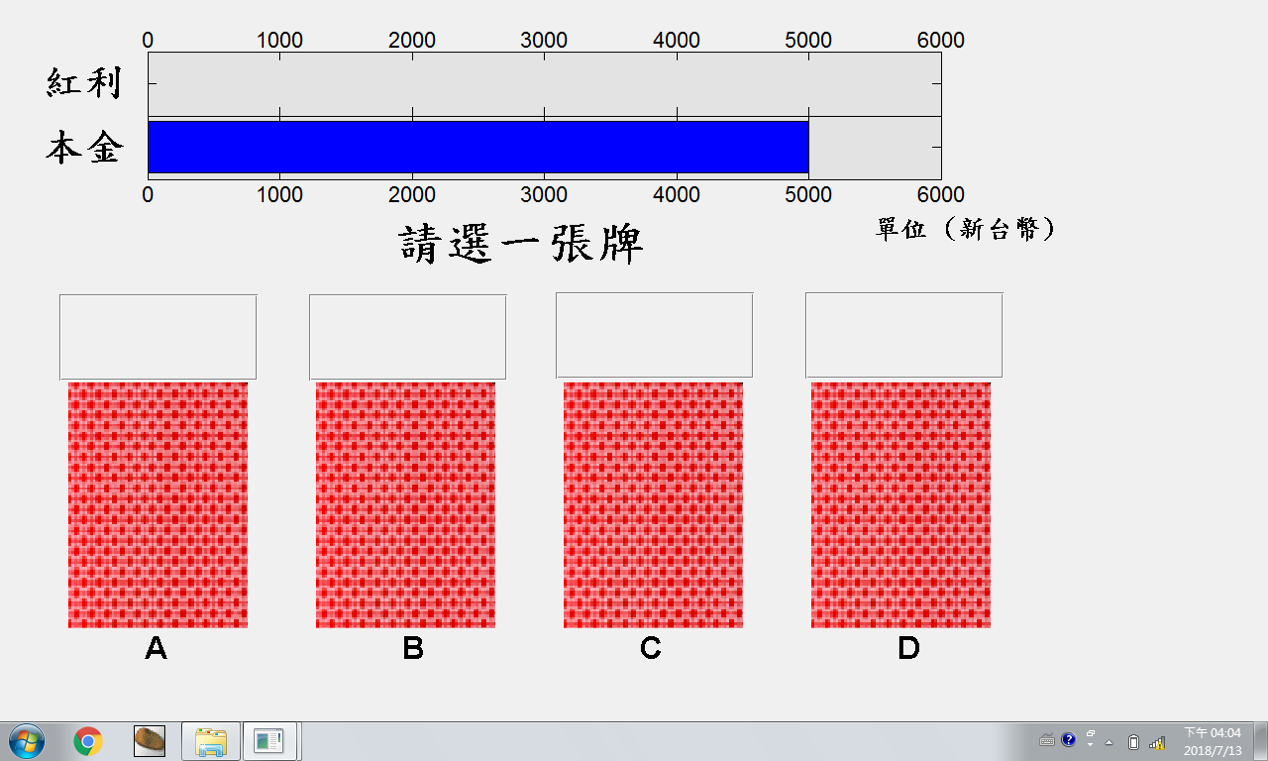


**Fig S1. Computerized IGT in traditional Chinese**

*Note:* The two horizontal bars at the top of the game screen respectively represent the extra money (upper bar) participants earned and the borrowed money from the bank (lower bar, in this version of IGT, the initial state of the borrowed money was setting as 5000 NT dollars). There are four decks at the bottom of the screen. In this game, participants can choose one deck for each trial. When the card is chosen (mouse button is pressed), after the card is turned over, the inner face of the card will be red or black, but the color has non-correlated with the final win or loss (Bechara et al., 1999; Bechara, 2007). The box above the deck will display the winning and losing money in each selection, and the gain or loss amount will be revealed on the top two bars of the accumulation.


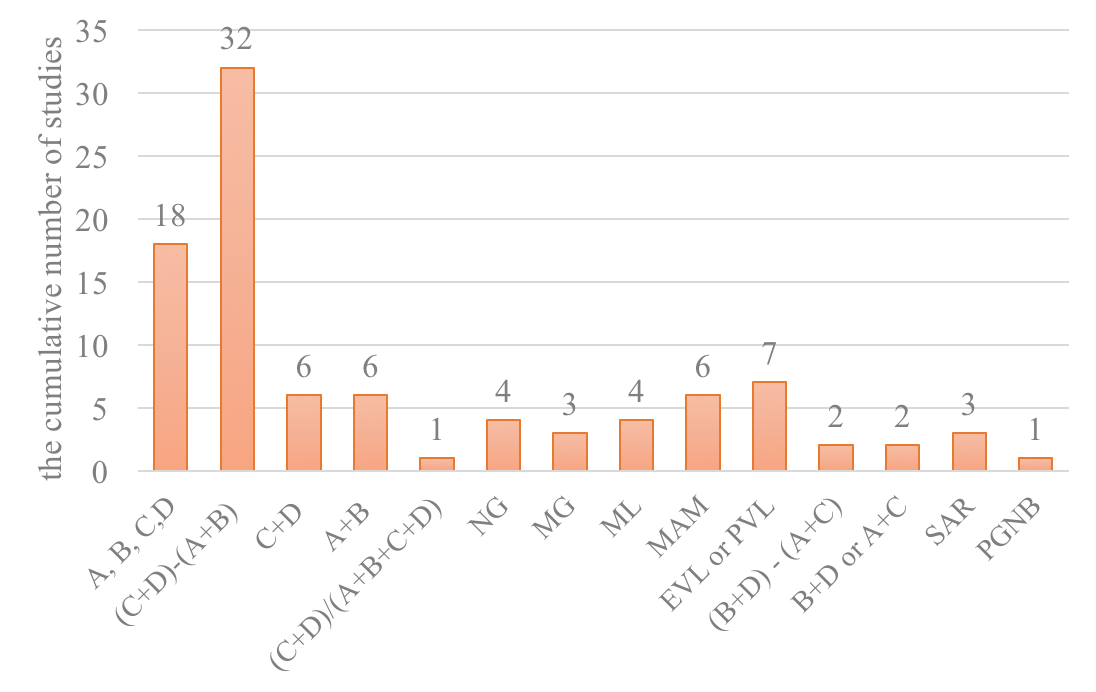


**Fig S2. IGT outcome measures in 38 studies**

*Note*: NG = net gain, MG = money gained, ML = money lost, EVL = expectancy valence model, PVL = prospect valence model, MAM = mean amount of money, SAR = subjective awareness ratings, PGNB = mean number of selections of previously-good-now-bad decks.


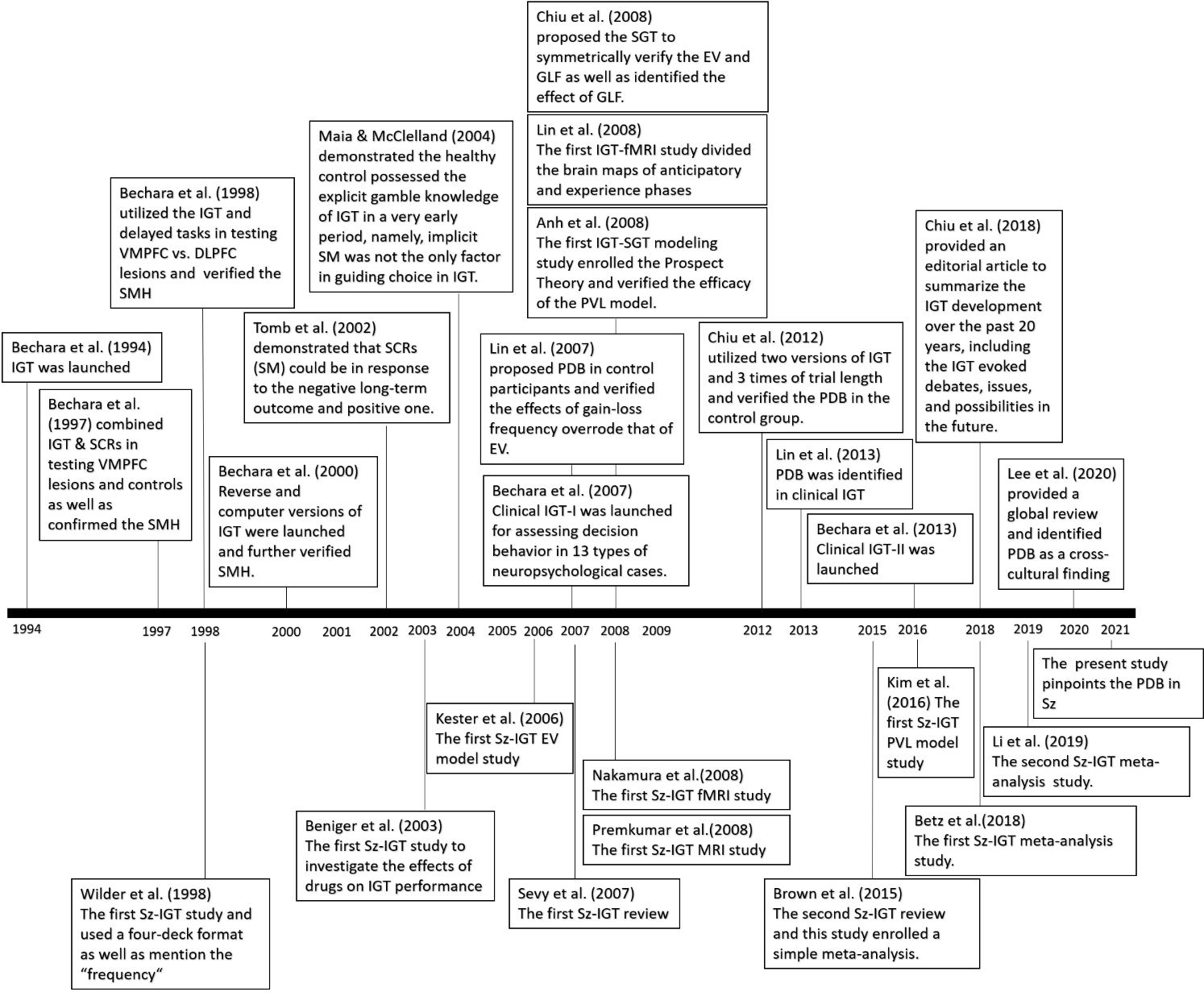


**Fig S3. IGT historical evolution map.**

*Note*: Here we depicted the development of global critical events of IGT and Sz-IGT-related events over the past 27 years. The upper panel depicted the global critical events of IGT and the lower panel depicted the critical studies of Sz-IGT related.

Sz=schizophrenia, SMH=somatic marker hypothesis, IGT=Iowa gambling task, EV = expected value, GLF = gain-loss frequency, VMPFC=ventromedial prefrontal cortex, DLPFC=dorsolateral prefrontal cortex, PVL = prospect valence model, MRI=magnetic resonance image, fMRI=functional magnetic resonance image.
